# Advancements in Radiology Report Generation: A Comprehensive Analysis

**DOI:** 10.3390/bioengineering12070693

**Published:** 2025-06-25

**Authors:** Dima Mamdouh, Mariam Attia, Mohamed Osama, Nesma Mohamed, Abdelrahman Lotfy, Tamer Arafa, Essam A. Rashed, Ghada Khoriba

**Affiliations:** 1 Center for Informatics Science, School of Information Technology and Computer Science (ITCS), Nile University, Giza 12588, Egypt; d.mamdouh2181@nu.edu.eg (D.M.); m.mohamed2133@nu.edu.eg (M.A.); m.osama2116@nu.edu.eg (M.O.); n.mohamed2126@nu.edu.eg (N.M.); alotfy@nu.edu.eg (A.L.); tarafa@nu.edu.eg (T.A.); 2 Graduate School of Information Science, University of Hyogo, Kobe 650-0047, Japan; 3 Advanced Medical Engineering Research Institute, University of Hyogo, Himeji 670-0836, Japan; 4 Faculty of Computers and Artificial Intelligence, Helwan University, Cairo 11795, Egypt

**Keywords:** radiology report generation, medical imaging, artificial intelligence, computer vision, natural language processing, transformers, graphs

## Abstract

The growing demand for radiological services, amplified by a shortage of qualified radiologists, has resulted in significant challenges in managing the increasing workload while ensuring the accuracy and timeliness of radiological reports. To address these issues, recent advancements in artificial intelligence (AI), particularly in transformer models, vision-language models (VLMs), and Large Language Models (LLMs), have emerged as promising solutions for radiology report generation (RRG). These systems aim to make diagnosis faster, reduce the workload for radiologists by handling routine tasks, and help generate high-quality, consistent reports that support better clinical decision-making. This comprehensive study covers RRG developments from 2021 to 2025, focusing on emerging transformer-based and VLMs, highlighting the key methods, architectures, and techniques employed. We examine the datasets currently available for RRG applications and the evaluation metrics commonly used to assess model performance. In addition, the study analyzes the performance of the leading models in the field, identifying the top performers and offering insights into their strengths and limitations. Finally, this study proposes new directions for future research, emphasizing potential improvements to existing systems and exploring new avenues for advancing the capabilities of AI in radiology report generation.

## 1. Introduction

Radiology is one of the most vital fields in medicine, focusing on the use of medical imaging to detect, diagnose, and aid in the treatment of diseases. It encompasses a variety of imaging modalities, including X-rays, Computed Tomography (CT), and Magnetic Resonance Imaging (MRI). To help interpret the findings in a radiological scan, radiologists make a report explaining the findings and any recommendations they wish to offer. Due to the rising demand for radiology services in recent years, there has been an increasing gap between the workload and the national radiologist workforce available [1]. This leads to significant delays in results being produced and increases pressure on radiologists, who cannot manage this increase in demand.

Radiology report generation (RRG) has emerged as a transformative artificial intelligence solution that directly addresses the mounting challenges in the field. Recent research highlights two primary approaches currently gaining prominence. Text summarization focuses on condensing existing radiology reports into concise, clinically relevant summaries by extracting key findings and critical information from lengthy diagnostic documents. In contrast, image-to-text generation directly converts medical images such as X-rays, CT scans, and MRIs into comprehensive diagnostic reports without requiring prior textual input. While text summarization processes existing textual data to create focused summaries, image-to-text generation interprets visual medical data to produce original diagnostic narratives. Both approaches leverage advanced AI techniques to enhance diagnostic efficiency and support radiologists in managing increased workloads, offering complementary solutions to the growing demand for radiology services.

Deep learning has led to great progress in text summarization for radiology reports, excelling at highlighting key medical details. Such approaches help to spot and pick out the most important points in radiology texts, making sure patient care summaries are correct and useful. A study by Liao et al. [2] reveals that deep learning is beneficial for generating radiology reports. Specifically, text summarization models demonstrate effectiveness across diverse work situations and datasets, enabling radiologists to improve efficiency and decrease report review time.

Significant achievements have been made in radiology report summarization by combining natural language processing (NLP) and deep learning. This has led to developing summaries that effectively highlight key medical findings. Applying these techniques allows critical information in radiology results to be collected accurately and made very valuable for the patients’ treatment. Sorting through unnecessary details, these systems make review easier, which helps radiologists pay closer attention to essential parts of the analysis. According to Nishio et al. [3], T5 models effectively generate automatic summaries, proving valuable to radiologists. The study reported that 86% of English reports and 85% of Japanese reports received positive feedback from radiologists. This method optimizes how work happens and ensures doctors have faster and smarter ways to make clinical decisions that boost patient health in urgent situations.

Beyond the capabilities of text summarization, image-to-text models in RRG offer a transformative approach by directly converting medical images into detailed, actionable reports. These models harness advanced deep learning techniques, such as Convolutional Neural Networks (CNNs) paired with recurrent neural networks (RNNs) or transformers, to generate comprehensive radiology reports from imaging data like chest X-rays or CT scans. They produce textual descriptions that include critical findings, impressions, and clinical recommendations, closely replicating the expertise of radiologists. A study by Miura et al. [4] introduces a novel method to enhance the factual completeness and consistency of image-to-text reports by incorporating reinforcement learning and semantic equivalence metrics like BERTScore, achieving a significant F1 score improvement in clinical information extraction, thereby improving the reliability of generated reports for clinical use.

RRG has demonstrated measurable improvements in report quality and diagnostic consistency. Automated systems can significantly reduce the workload of radiologists and accelerate the diagnostic process, an essential advantage in light of the global shortage of radiology professionals and the increasing volume of imaging data [5]. RRG systems have also shown strong potential in improving radiological reports’ consistency and clinical quality, with several advanced models now achieving performance levels comparable to human experts in accuracy and clinical relevance [6].

For instance, the MedVersa model achieved RadCliQ-v1 scores 1.46 ± 0.03 on the IU X-ray findings sections, outperforming other AI systems in clinical relevance metrics [7]. Comparative studies further reveal that AI models can surpass radiologists in certain diagnostic tasks, with higher AUROC (Area Under the Receiver Operating Characteristic) scores (0.91 vs. 0.86) and the detection of 6.8% more clinically significant findings [8]. Beyond accuracy, AI-driven report generation enhances diagnostic precision, report diversity, and nuanced communication, critical elements for patient care and clinical decision-making [6]. Quality assessments of AI-generated reports reflect this improvement: radiologists rated summary quality at 4.86/5 and recommendation agreement at 4.94/5, while patient comprehension scores nearly doubled, from 2.71 to 4.69/5, when layperson-friendly AI reports were used [9].

**Figure 1:**
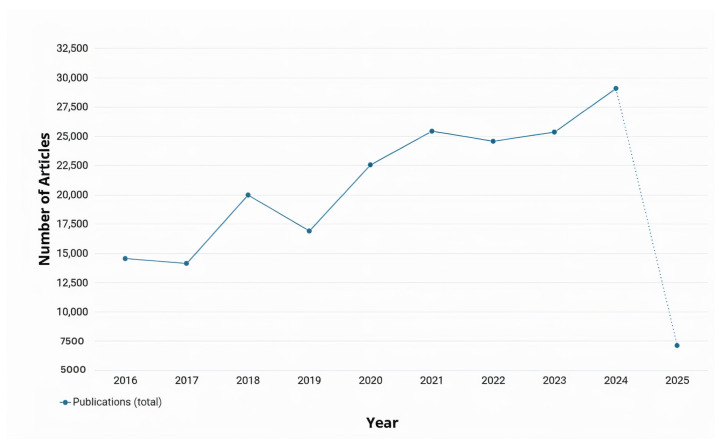
Total number of annual publications related to RRG from 2016 to 2025 [19]. However, the 2025 data is incomplete; it is included as dotted to indicate the continuation of the timeline.

**Figure 2:**
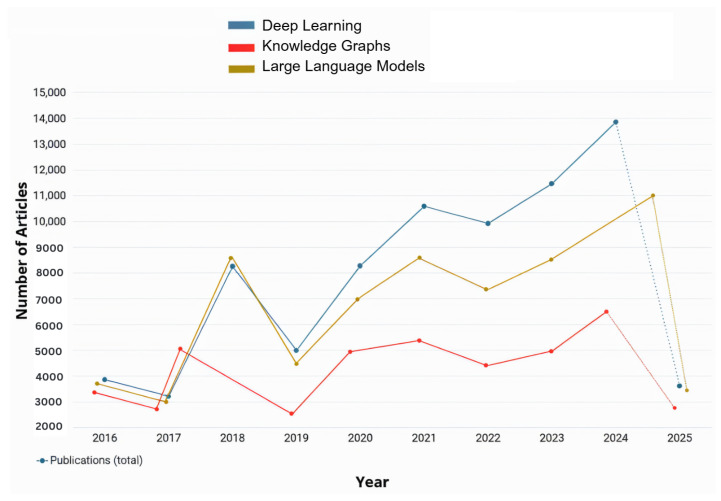
Annual number of publications related to RRG using deep learning, Large Language Models, and knowledge graphs as retrieved from Dimensions.AI. The data includes results from 2016 to 2025.

In the past decade, numerous researchers have contributed to advancing RRG and its outcomes by applying various AI techniques as demonstrated in Figure 1. In recent years, there have been several surveys on RRG [10,11,12]. These surveys aimed to explore the advances in computer vision (CV) and NLP within the RRG domain and their impact on its development. Recently, a surge of studies aimed to evaluate the performance of Large Language Models (LLMs), such as GPT-3.5, in summarizing radiology reports or generating them from scratch based on radiological images [13,14,15,16,17,18]. The bibliometric data in Figure 1 and Figure 2 were retrieved from the Dimensions AI platform [19]. For Figure 1, the search query was “Radiology Report Generation” across publication years 2016–2025. For Figure 2, this base query was refined to focus on specific AI techniques: deep learning, Large Language Models, and knowledge graphs. Finding new and more efficient ways for automated RRG is still an ongoing area of research that has the potential to alleviate the workload on radiologists significantly. It can also help inexperienced radiologists by acting as a second reader and an automatic tool for flagging any potential abnormalities in an image immediately [10].

**Figure 3:**
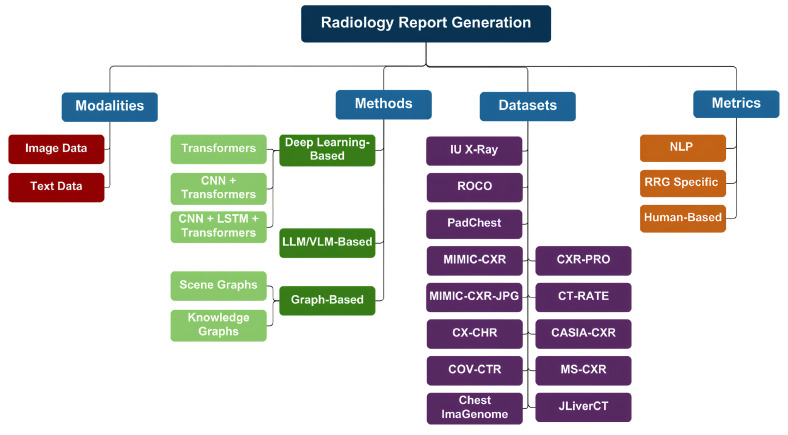
Overview of our RRG survey. The diagram outlines input modalities (chest X-rays, clinical text), categorizes methods (deep learning, large pre-trained models, and graph-based techniques), lists common datasets, and details evaluation metrics (NLP scores, clinical measures, and human review).

To ensure a comprehensive and relevant survey of recent advancements in RRG, we conducted a structured search across multiple scientific databases. The databases and platforms included IEEE Xplore, Springer Nature Link, ScienceDirect, arXiv, MDPI (Multidisciplinary Digital Publishing Institute), and major computer vision and AI conference proceedings such as CVPR (Conference on Computer Vision and Pattern Recognition), ICCV (International Conference on Computer Vision), and AAAI (Association for the Advancement of Artificial Intelligence). Additionally, Google Scholar was used to capture articles from diverse sources and preprints not indexed in traditional databases. A total of 38 peer-reviewed papers were selected based on their focus on deep learning, vision-language models (VLMs/LLMs), and graph-based methods for RRG. The inclusion criteria prioritized publications from 2022 through 2025 that proposed novel architectures, benchmarking strategies, or clinically relevant evaluations for automated radiology reporting. We will also discuss the methods used for RRG, the most prominent datasets available, the evaluation metrics of generated reports, and the limitations present in the current research, and offer suggestions for future studies. An overview of our paper can be seen in Figure 3.

This paper will be organized as follows: Section 2 will discuss RRG and its various methods. In Section 3, we will review the datasets used for RRG. Afterward, in Section 4, we will focus on the evaluation metrics used for RRG. Section 5 will focus on the recorded model performance results in RRG. Section 6 will outline the current open issues in RRG research. Finally, we will offer some suggestions for future studies in Section 7.

## 2. Radiology Report Generation

By combining the capabilities of CV and NLP, AI systems can analyze complex radiological images and extract key information from reports. This synergy enables the development of AI-driven solutions that automatically generate comprehensive and detailed reports. Such advancements significantly reduce the time required to produce accurate reports, highlighting critical findings and identifying abnormalities. This paper reviews the most prominent applications and studies on RRG using AI. These applications are categorized into three main approaches: deep learning-based methods, LLM/VLM-based methods, and graph-based methods, each of which will be examined in detail. Summary tables for each category are provided after each section.

### 2.1. Deep Learning-Based Methods

The use of deep learning methods for RRG marked the beginning of AI’s integration into medical imaging, particularly in analyzing radiological images. Over the past decade, numerous studies have explored various deep learning tools for RRG, aiming to evaluate their accuracy and efficiency as illustrated in Figure 2. In the context of RRG, deep learning primarily involves Convolutional Neural Networks (CNNs), recurrent neural networks (RNNs), and encoder-decoder architectures. These models are designed to automatically extract visual features from medical images and generate corresponding textual descriptions in the form of radiology reports. Typically, a CNN-based image encoder processes the input image to generate a feature representation, which is then passed to a language decoder, often implemented using RNNs, LSTMs, or Transformers, to produce the report. Transformer-based models have grown very popular nowadays, as they are able to model both longer and more complicated relationships better than other models. Most of these models apply ViTs (Vision Transformer) for turning images into estimates, use multi-head attention to pick out important picture details, and set up encoder-decoder structures to turn images into language. Many people use BERT (Bidirectional Encoder Representations), CLIP (Contrastive Language–Image Pre-training), and BioBERT to perform text encoding and align semantic meanings. We focus, in the following parts of this section, on how certain models change or add to these standard parts to handle specific problems in radiology report generation.

#### 2.1.1. Transformers-Only Approaches

The METransformer, proposed by Wang et al., adopts a Vision Transformer (ViT) in its architecture to encode radiographic images using several learnable expert tokens [20]. These expert tokens, such as “multi-specialist diagnoses”, act as independent attention heads that focus on different image regions, capturing fine-grained visual details. Rather than repeating standard transformer mechanisms, the model introduces a multi-expert diagnostic framework in which each token specialized in specific features. A bilinear attention encoder refines the interactions between the image features and expert tokens, and a multi-expert bilinear attention decoder aggregates their outputs into a single, high-accuracy report.

Wang et al. proposed the Medical Semantic-Assisted Transformer, which introduced a memory-augmented sparse attention mechanism into an encoder–decoder architecture [21]. The encoder extracts features from images using a pre-trained CLIP model, which are then passed into a memory-augmented sparse attention (MSA) block to model higher-order interactions with memory tokens containing clinical context. The model incorporates a Medical Concept Generation Network (MCGN) to identify and embed essential medical concepts, enhancing the integration of clinical knowledge into the generated reports. The decoder merges visual and conceptual embeddings, with sparse attention mechanisms focusing on relevant content.

The Knowledge-Injected U-Transformer (KiUT) by Huang et al. provides a variation in the cross-modal U-Transformer architecture for generating descriptive radiology reports [22]. This architecture connects the image–text modalities through a U-shaped structure that effectively facilitates information exchange between the encoder–decoder layers. The KiUT architecture comprises the Cross-modal U-Transformer, the Injected Knowledge Distiller, and the Region Relationship Encoder. The Cross-modal U-Transformer applies multi-head self-attention to extract key visual features, which are subsequently consolidated by a decoder using U-connections. U-connections allow refined image representations to flow into the Knowledge Distiller. The Injected Knowledge Distiller merges multi-source visual, contextual, and clinical information. It utilizes image features to provide the encoder with visual knowledge, extracts contextual knowledge from medical texts using a pre-trained MBERT (Multilingual BERT) model fine-tuned on clinical data, and incorporates clinical knowledge by encoding a graph of clinical symptoms linked through the Graph Attention Network (GAT), which captures real-world symptom relationships. The Region Relationship Encoder supports spatial understanding by modeling the spatial geometry of image regions. This enables the model to recognize intrinsic and extrinsic relationships within these regions, resulting in diagnostically relevant and highly context-sensitive reports.

CT2Rep, proposed by Hamamci et al., directly addresses the challenges posed by volumetric data from CT scans in generating radiology reports using 3D medical imaging [23]. It includes a 3D vision feature extractor followed by a transformer encoder and decoder equipped with relational memory and memory-driven conditional layer normalization (MCLN). The feature extractor segments CT volumes into latent space patches while preserving spatial structure. These are processed by dual transformers for spatial and latent dimensions. The relational memory enables pattern retention across sequences, and MCLN enhances contextual awareness during report generation. An extended version, CT2RepLong, uses cross-attention to incorporate prior imaging and report history, supporting longitudinal report generation across multiple patient visits.

Sirbu et al. proposed GIT-CXR [24], a transformer-based end-to-end architecture for RRG, leveraging the Generative Image-to-text Transformer (GIT) model initially designed for general vision-language tasks such as image/video captioning and question answering [25]. Their framework uses a pure transformer pipeline, incorporating a vision encoder and a text decoder to generate clinically coherent reports directly from chest X-ray images. The architecture explores both single-view (SV) and multi-view (MV) imaging, where the latter incorporates multiple imaging perspectives (e.g., AP and Lateral) to enhance visual representation. Chest X-ray images are first processed by the vision encoder, which extracts high-dimensional visual embeddings from the images. In the MV imaging, temporal embeddings are added to distinguish between different imaging perspectives (e.g., AP and Lateral), and these are concatenated to form a unified representation. To further improve performance, they incorporated contextual information (C) from patient history and indication into the transformer decoder, which sequentially generates the textual report. Additionally, an auxiliary multi-label classification head is attached to the encoder to predict pathology labels during training. Most notably, they implemented a curriculum learning (CL), a strategy that gradually increases report complexity during training by starting with shorter, simpler report samples and progressing to longer, more difficult ones. This strategy led to significant improvements in generating longer, diagnostically rich reports. Adding this complexity helps the model better capture detailed patterns in extended diagnostic descriptions.

#### 2.1.2. CNN and Transformer Hybrid Approaches

Posterior-and-Prior Knowledge Exploration and Distillation (PPKED) was a framework introduced by Liu et al. that achieved synergy between multiple components [26]. In the PPKED framework, ResNet-152 was used as a feature extractor alongside three other modules: the Posterior Knowledge Explorer (PoKE), the Prior Knowledge Explorer (PrKE), and the Multi-domain Knowledge Distiller (MKD). The PoKE identified abnormalities by mapping features to disease topics via a “topic bag”, where predefined abnormalities, such as cardiomegaly and emphysema, directed the model’s attention to specific areas. This process imitated the labeling of particular image regions for diseases while filtering out irrelevant topics in the diagnostic process. The PrKE utilized knowledge from past radiology reports as historical context, creating BERT embeddings for cosine similarity matching to align current findings with past cases. The MKD integrated all these components into a coherent report, adaptively weighted by the Adaptive Attention (ADA) mechanism. This mechanism selectively extracted pertinent information from both posterior and prior knowledge sources, resulting in accurate and contextually rich reports.

Another application is the CNX-B2 model by Alqahtani et al., which integrates CNN and transformer with a ConvNeXt-based image encoder and BioBERT decoder [27]. ConvNeXt is an advanced CNN architecture with transformer-inspired enhancements that capture intricate visual details through large kernel sizes and inverted bottlenecks for rich and expressive image embeddings. These are then passed to BioBERT, which has been adapted with cross-attention mechanisms for report generation. Such a setup allows BioBERT to effectively map the visual features to medical language so that the reports are detailed and relevant for a medical context. The combination of ConvNeXt and BioBERT guarantees that there will be some visual information that precisely corresponds to medical language when generating cytological reports.

Moreover, TranSQ, proposed by Gao et al., simulated the workflow of a radiologist in generating reports and segmented the process into three major tasks: comprehension of the visual properties, intention based on observation, and the generation of descriptive sentences [28]. TranSQ relied on three fundamental modules: a ViT-based visual extractor, a semantic encoder, and a report generator. When segmenting medical images into patches, the visual extractor used type and spatial embeddings to capture positional information. The transformer encoder modeled relationships between each image using multi-head self-attention, enabling a holistic representation of the visual features. To focus on relevant visual information, the semantic encoder projected these features using learnable embeddings as queries, referred to as observation intentions. Subsequently, multi-head cross-attention correlated the visual features with observation intentions, ensuring that the generated semantic features were specific to the medical findings. These characteristics were then fed into a transformer-based report generator, which, together with retrieval and generation-based strategies, used MCLN to produce coherent medical sentences. TranSQ employed a multilayer perceptron to select text and organize the report within the conventions of radiology language, thereby increasing the accuracy and interpretability of the generated text.

Cao et al. developed a Multimodal Memory Transformer Network (MMTN) that generated semantic reports by fusing image features with terminological embeddings [29]. MMTN was constructed with a multimodal fusion layer and an encoder–decoder architecture. The encoder processed the image by segmenting it into regions of equal size, extracted the grid features using a pre-trained CNN, and combined them with the terminological embeddings from a BERT-based model trained on medical terms relevant to gastrointestinal and thoracic diseases. This alignment contextualized the specific findings and enhanced the relevance of the report. The enriched features in the decoder combined with the previously generated words to form semantic states. Successive layers incorporated visual features through multi-head attention, refining the report content. Finally, the multimodal fusion layer merged visual and linguistic information to emphasize the essential findings. By aligning medical terminologies with image features through memory-augmented attention, MMTN generated clinically comprehensive and contextually accurate reports.

Another application was the interactive and explainable region-guided radiology report generation by Tanida et al., which modeled the radiologist’s region-focused examination [30]. The model incorporated a Faster R-CNN detector with a ResNet-50 backbone to extract and classify specific anatomical regions. A binary classifier identified regions warranting description, while an abnormality classification module flagged potentially pathological areas. For report generation, a fine-tuned GPT-2 model conditioned the output on visual features and previously generated tokens, utilizing pseudo-self-attention to inject visual data into the text generation process. This multi-step approach enabled the model to produce precise, structured reports guided by normal and abnormal findings, closely mirroring the radiologists’ diagnostic workflows.

At the same time, Onakpojeruo et al. [31] introduced a new Conditional Deep Convolutional Neural Network (C-DCNN) and studied its capacity by classifying brain tumors in regular MRI data and those generated by GANs. Since there is not enough real data available and privacy is an issue in medical imaging, the authors used a Deep Convolutional Generative Adversarial Network (DCGAN) to make alternative images of brain and kidney tumors. The datasets obtained were trained in the C-DCNN, a model with enhanced abilities, including dropout layers, attention blocks, residual transformation, and layer normalization to aid in keeping errors small and model versatility. Several top-performing networks were compared with the proposed model, and it was demonstrated that the new model had higher scores on important measures. In particular, the authors argued that the GAN-made data were useful diagnositically useful as the original kinds and also preserved the patients’ anonymity, showing how significant this approach is for developing better models. It demonstrates that synthetic data are useful for improving accuracy and can be used to avoid privacy issues in radiology work. Using solid visual feature extractors during radiology report generation helps improve the dependability and accuracy of the descriptions included in the reports.

Lang et al. proposed a Dual Attention and Context Guidance (DACG) model that addresses two key challenges in the field: visual–textual data bias and long-text generation [32]. Their architecture uses a ResNet-101 network to extract visual features from chest X-ray images, which are then refined using a Dual Attention Module (DAM). The DAM is composed of two components—a Position Attention Block (PAB) that captures spatial dependencies across image regions, and a Channel Attention Block (CAB) that models inter-channel relationships—allowing the model to better identify and focus on subtle or localized abnormalities. To further enhance report generation, a Context Guidance Module (CGM) is integrated into the decoder. The CGM includes a guidance memory that records relevant textual descriptions from previous steps and feeds this contextual information into a modified normalization layer, enabling the model to generate longer, coherent, and clinically rich narratives. This design ensures more accurate highlighting of abnormal findings and better alignment between image features and generated reports.

#### 2.1.3. CNN, Transformers and LSTM Combined Approaches

Contrastive Attention for Automatic Chest X-ray Report Generation by Liu et al. combined a ResNet-50 with a hierarchical LSTM and a novel mechanism, Contrastive Attention [33]. The proposed approach contrasts an input X-ray image against a so-called “normality pool” to highlight abnormal regions by employing Aggregate Attention. This mechanism identifies similar typical images and differentiates attention to emphasize unique abnormalities. By doing so, the model enhances its focus on clinically relevant regions and generated reports that prioritize abnormal findings in diagnostic areas.

Moreover, Zhou et al. presented Visual–Textual Attentive Semantic Consistency for Medical Report Generation, which uses DenseNet-201 combined with multimodality semantic attention and an LSTM-based sentence decoder [34]. DenseNet-201 extracts region-specific features that are further refined through semantic attention to disease characteristics, such as the location and size of lesions. Clinical context is integrated using BioSentVec embeddings, which represent patient demographics and medical history. This attention model links visual, clinical, and label-based features with the LSTM sentence decoder to produce coherent text in real-world radiology reports, ensuring both linguistic and diagnostic consistency.

Tsaniya et al. proposed an Automatic Radiology Report Generator Using a Transformer with Contrast-Based Image Enhancement, which describes a structured pipeline for improving the quality of radiology reports generated from X-ray images by enhancing the images before processing [35]. The pipeline begins with image enhancement techniques, including histogram equalization, CLAHE (Contrast Limited Adaptive Histogram Equalization), EFF (Exposure Fusion Framework), and Gamma Correction, to make critical features more visible. These enhanced images are then processed by ChexNet, which uses a DenseNet121-based image encoder to extract feature vectors that preserve key details. Simultaneously, the model generates BERT embeddings to provide contextual meaning to the medical terms in the report. Multi-head attention aligns image and text features into a unified context vector that captures meaningful relationships between visual data and semantic content. Additionally, the model employs an LSTM decoder, where the gated structure allows it to retain and utilize crucial contextual information for coherent sentence generation. BERT embeddings further guide word prediction to ensure the selection of appropriate medical terms. This approach proves particularly useful for diagnostic imaging applications requiring enhanced sharpness, such as mammography and orthopedic radiography, resulting in comprehensive and contextually accurate reports generated from improved image inputs.

The work of Onakpojeruo et al. [36] is important for the CNN-based pipeline in radiology because they use the Pix2Pix-based augmentation framework with GAN to help classify brain tumors from MRI data. They have a strategy that importantly focuses on medical imaging AI: the limited number of datasets and the danger of privacy issues. To add to the real data, the authors made a large amount of synthetic images using high-quality brain MRI images of glioma, meningioma, pituitary tumors, and typical brain samples. The pictures were provided to develop the CDCNN, which was further enhanced with residual connections, attention mechanisms, and dropout regularization, helping it perform better and improve stability during the learning process. With the help of the new synthetic data, model accuracy, F1 scores, and AUC values saw certain increases in all tumor types, showing how accurate the additions are. It shows how using GAN-based synthetic images in radiology reports is valuable when GANs are trained on unique datasets that raise the accuracy of visual feature extractors, such as CNNs, so their results feed the transformers used for report writing and the knowledge graphs used in the system. Particularly, by relying on a Pix2Pix conditional GAN, modeled after U-Net architecture, and adopting PatchGAN discriminators, the algorithm guarantees that the generated images are accurate at the pixel level and appear lifelike, so it is appropriate for both vision-language and multimodal AI work.

The Image-to-Indicator Hierarchical Transformer for Medical Report Generation (IIHT), proposed by Fan et al., emulates diagnostic workflows by conditioning report generation on hierarchical disease indicator classification and expansion [37]. A pre-trained ResNet serves as a feature extractor for the images, while a bidirectional GRU expands these features into disease-specific textual sequences that the model re-encodes as indicators. The transformer-based generator then utilizes these indicators, along with multi-head self-attention applied to both visual and textual contexts, to produce the final report. This approach ensures that the generated reports align with the structure and language of clinical diagnoses, effectively mirroring an in-depth, clinician-like reasoning process. A summary of the models discussed above can be seen in Table 1.

### 2.2. VLM- and LLM-Based Methods

As observed in Figure 2, the number of applications and studies conducted about RRG implemented or integrated with LLMs or vision-language models (VLMs) has risen significantly over the past two years and is expected to continue increasing. This growth is driven by the ongoing advancements in LLM and VLM architectures, which provided powerful tools for RRG. This trend is further explored in the following section, where some of the most recent studies on the utilization of LLMs/VLMs for RRG are reviewed. LLMs are pre-trained on large corpora of general and domain-specific text, enabling them to understand complex language structures and produce coherent and contextually accurate narratives. When applied to RRG, LLMs can be fine-tuned or prompted to generate radiology reports from either structured findings or embedded image features. On the other hand, VLMs use pre-trained vision encoders (e.g., CNNs or Vision Transformers) to extract image features and then align them with the text generation capabilities of an LLM. This integration allows the model to capture both visual pathology and relevant clinical context, enabling more accurate, fluent, and interpretable radiology reports.

Voinea et al. explored the potential of LLMs in automating RRG, focusing on fine-tuning the Llama 3-8B model, a variant within Meta’s Llama series [38]. Their research evaluated whether a fine-tuned model, trained on a radiology-specific dataset, could enhance medical diagnostics by generating accurate and contextually appropriate clinical conclusions. Fine-tuning was employed to adapt the pre-trained model to the unique linguistic, stylistic, and contextual demands of medical diagnoses, enabling it to produce meaningful and precise findings. Traditionally, radiologists invested significant time and cognitive effort in analyzing medical data to derive concise and actionable summaries. By leveraging the capabilities of LLMs, the authors aimed to alleviate this workload and provide a robust tool to streamline the generation of radiology report conclusions.

Pellegrini et al. introduced the RaDialog architecture, which integrates VLMs with vision encoders (e.g., BioViL-T) and language decoders (e.g., GPT variants) for multimodal RRG [39]. By combining image features from chest X-rays with structured text inputs using multimodal fusion, RaDialog produces accurate, clinically relevant, and context-sensitive reports. Its instruct-tuning capability allows interactive adjustments, such as incorporating user feedback and refining language for specific audiences. Additionally, the CheXpert findings classifier improved pathology predictions, ensuring high clinical accuracy. RaDialog’s multimodal and interactive features streamlined radiology workflows, making it a transformative tool for generating tailored and efficient medical reports.

Liu et al. proposed an adaptation of the MiniGPT-4 model for RRG. Their architecture introduced two key components—In-domain Instance Induction (I3) and Coarse-to-Fine Decoding (C2FD)—which aimed to enhance the model’s ability to generate accurate and domain-specific radiology reports from chest X-ray images [14]. The I3 module adapted the general-purpose MiniGPT-4 to the medical field by fine-tuning it with annotated radiology image–text pairs, enabling the model to learn medical terminology, anatomical structures, and diagnostic patterns effectively. The C2FD module improved coherence and clinical precision in reports through a two-step process: it generated a coarse draft from input images and then refined it by correcting errors, removing irrelevant details, and enhancing clinical accuracy. When combined with I3 and evaluated on the IU X-Ray and MIMIC-CXR datasets, this approach significantly enhanced report quality and enabled the model to generalize effectively across diverse medical scenarios. The combination of I3 for domain adaptation and C2FD for report refinement substantially improved the accuracy and relevance of the generated reports. The architecture enhanced the model’s comprehension of medical terminology, anatomical details, and diagnostic patterns, resulting in precise and coherent outputs. Furthermore, the C2FD module refined report quality by correcting errors and filtering irrelevant content, offering reliable and valuable outputs for healthcare professionals. This approach also demonstrated the potential to adapt general-purpose LLMs to specialized domains with limited data, providing a practical framework for similar applications.

In the field of AI for medical applications like RRG, a critical challenge has been the occurrence of fabricated content in AI-generated reports that does not align with real-world medical data. To address this issue, Zhang et al. developed RadFlag, a black-box hallucination detection model [40]. RadFlag uses an entailment-based evaluation approach to compare generated reports with ground-truth medical findings, categorizing content as fully, partially, or not supported by the input data. This process aims to ensure that diagnoses and observations in generated reports are grounded in the provided evidence. The model’s performance is measured using entity precision and relation precision, which assesses its ability to accurately identify and relate medical concepts in reports. To evaluate RadFlag, Zhang et al. tested it on two state-of-the-art RRG models, Medversa and RaDialog, using chest X-ray datasets with paired medical images and reports. The experiments demonstrated that RadFlag effectively detected hallucinated findings by cross-checking generated content against established medical knowledge. This improved the reliability of automatic RRG systems, making RadFlag a valuable tool for enhancing the quality and trustworthiness of AI-generated medical reports.

Udomlapsakul et al. proposed an advanced system for RRG using a domain-specific multimodal language model tailored for interpreting chest X-rays and producing clinically relevant reports [41]. The system followed a two-stage approach. First, the model was pre-trained on the LLaVa-MED multimodal medical dataset, augmented with a randomly initialized adapter to embed domain-specific medical knowledge. This pre-training provided a strong foundation in medical contexts and terminologies. Next, the model was fine-tuned on the interpret-cxr dataset, containing over one million image–text pairs. To enhance efficiency and accuracy, the dataset was refined to include only the first X-ray image from each study, resulting in a diverse set of 700,000 image–text pairs. The authors addressed challenges in radiology report datasets, which often contain extraneous details such as dates and doctor names. Initially, GPT-3.5 Turbo was used to clean the dataset by removing irrelevant information. However, this cleaning led to performance inconsistencies due to mismatches with uncleaned test datasets. As a result, the model was trained on the original uncleaned dataset. Two innovative prompts were introduced to improve output quality: the “Report Refinement” prompt, which enhances readability and clarity, and the “First, Do No Harm” SafetyNet, which leverages the X-Raydar classifier to identify and rectify potential errors. These strategies significantly enhanced the model’s ability to generate accurate and contextually reliable reports, demonstrating its potential as a valuable AI tool for clinical decision-making.

Li et al. introduced Knowledge-enhanced Radiology Report Generation (KARGEN), a framework that combined LLMs with a medical knowledge graph to produce detailed and clinically accurate radiology reports [42]. The core innovation of KARGEN is its use of a Graph Convolutional Network (GCN) to incorporate domain-specific knowledge from a medical knowledge graph. This knowledge was fused with visual features extracted from chest X-ray images using a pre-trained Swin Transformer, a robust architecture designed for vision tasks. LLaMA2-7B was used for report generation, integrating fused features from X-ray images and disease-specific knowledge. KARGEN was trained and evaluated on the MIMIC-CXR and IU-Xray (Indiana University X-ray dataset) datasets, which provided extensive paired chest X-ray images and radiology reports. By combining image-based and knowledge-based features, KARGEN constructed nuanced and contextually sensitive representations of medical images, enabling it to generate linguistically accurate and medically informative reports.

Srivastav et al. introduced Multimodal AI for Radiology Applications (MAIRA), a large multimodal model designed to generate radiology reports from chest X-ray images [43]. MAIRA built upon the MAIRA-1 architecture and integrated both frontal and lateral image views to enhance the accuracy and comprehensiveness of its reports. It utilizes a RAD-DINO-like image encoder, optimized for medical imaging within the radiology domain, and was trained in a multitask setup to generate detailed findings and summary impressions. The model was trained and evaluated using multiple public datasets, including MIMIC-CXR (Medical Information Mart for Intensive Care Chest X-ray dataset), CheXpert, PadChest, BIMCV-COVID19, and Open-I, which provided a diverse collection of X-rays and radiology reports for benchmarking and training.

Additionally, MAIRA employed GPT-4 to produce multiple candidate reports, selecting the most accurate and comprehensive one, effectively minimizing hallucinated or incomplete information. The architecture’s scalability enabled larger versions, such as Vicuna-7B and Vicuna-13B, to improve performance while maintaining competitive results with smaller-scale models. Wang et al. introduced History-Enhanced Radiology Report Generation (HERGen), an advanced AI-driven framework designed to streamline and accelerate RRG from medical images [44]. By integrating cutting-edge CV and NLP technologies, HERGen generated detailed, accurate, and clinically relevant reports. The framework utilized state-of-the-art models like DistilGPT2 for text generation and CXR-BERT for understanding medical language, ensuring high-quality outputs. One of HERGen’s most innovative features was its ability to perform longitudinal studies by analyzing sequences of radiographs taken over time. This capability allowed the framework to track disease progression or improvement, assisting radiologists in efficiently reviewing a patient’s medical history. HERGen also minimized the risk of missing critical details in medical images and provided consistent, reliable reports, making it an invaluable tool for augmenting manual reporting processes.

Alkhaldi et al. introduced MiniGPT-Med, a state-of-the-art multimodal AI model designed for radiology diagnostics [45]. This model combines the strengths of LLMs and ViTs to tackle complex medical vision-language tasks. MiniGPT-Med excels in generating radiology reports, detecting diseases, and answering medical questions based on imaging data. Its architecture integrates vision and language components, utilizing large pre-trained vision-language models fine-tuned on specialized medical datasets. A key dataset used in its training is MIMIC-CXR, which provided over 200,000 chest X-ray images paired with radiology reports. This dataset enables the model to learn intricate relationships between visual features and textual descriptions, resulting in accurate and contextually appropriate outputs. MiniGPT-Med outperforms both specialist and generalist models in tasks such as abnormality detection and report generation, achieving state-of-the-art performance. It demonstrates proficiency in grounding tasks, like associating specific abnormalities with descriptive text, and non-grounding tasks, such as generating free-form radiology reports.

Kumar et al. described Flamingo-CXR, a VLM designed to process both visual and textual data for generating radiology reports from chest X-ray images [6]. Based on the Flamingo architecture, the model utilizes a pre-trained transformer fine-tuned specifically for medical report generation tasks. Flamingo-CXR combines a vision encoder to extract features from X-ray images and a language decoder to translate these features into coherent, human-like radiology reports. Its zero-shot learning capability allows it to adapt to diverse clinical scenarios without requiring explicit training for every condition. Flamingo-CXR was trained on large-scale datasets like MIMIC-CXR, which provide labeled chest X-rays paired with radiology reports, and IND1, a dataset reflecting diverse patient populations in India. These datasets cover a wide range of pathologies, including pneumonia, cardiomegaly, and pulmonary edema, forming a robust foundation for the model’s training. Additionally, Flamingo-CXR benefits from fine-tuning with expert radiologist feedback to ensure clinical accuracy. However, it faced challenges with rare or out-of-distribution conditions, requiring additional fine-tuning with specialized datasets to address such limitations.

Kapadnis et al. introduced SERPENT-VLM, a multimodal framework for generating accurate radiology reports from chest X-rays [46]. Combining a frozen visual encoder with a LLM, SERPENT-VLM processes radiographic images into high-dimensional feature representations, which the LLM uses to generate initial reports. To improve accuracy, the model employs a novel self-refining loss mechanism, iteratively aligning generated reports with relevant image features and addressing inaccuracies, particularly in noisy or low-quality images. SERPENT-VLM was evaluated on the benchmark datasets IU-Xray and ROCO(Radiology Objects in COntext dataset), which included noisy and diverse radiographic images paired with reports. The model demonstrated resilience to variable image quality and successfully generated contextually grounded reports. An attention-based aggregation mechanism ensured focus on the most relevant image regions, reducing hallucinations and enhancing diagnostic reliability.

Campanini et al. developed iHealth-Chile-1, a multimodal architecture for RRG that combines advanced vision encoders (e.g., CLIP and BiomedCLIP) with language models (e.g., LLaMA and Vicuna) [47]. The vision encoder extracts features from chest X-rays, while the language model generates detailed textual reports. BiomedCLIP, trained exclusively on medical data, enhances the system’s precision in interpreting chest X-rays. The report generation process involves two stages: training on image data to extract key features and refining reports using enriched prompts with predefined templates and a DenseNet-121 classifier. These prompts structured findings into concise, contextually accurate reports, though they introduced trade-offs, such as a decline in pathology classification metrics like F1-cheXbert. The model was trained on datasets like CheXpert, which provided annotated chest X-rays and detailed reports on conditions like pneumonia and fractures.

Hou et al. proposed RADAR, a two-stage framework for radiology report generation that integrates both the internal knowledge of LLMs and externally retrieved domain-specific information [48]. In the first stage, a multimodal LLM such as BLIP-3 processes the chest X-ray image along with clinical prompts to produce preliminary findings. These generated statements are then filtered using an expert observation classifier, built on DenseNet-121 and trained to recognize clinical observations from images, to extract only the high-confidence observations. In the second stage, RADAR enhances the report by retrieving supplementary findings from a structured database of similar annotated radiology cases, using a KL-divergence-based similarity measure. Only findings that are complementary (i.e., not already captured by the internal model) are integrated into the prompt, which is then passed again to the LLM to generate the final report. This architecture allows RADAR to leverage the reasoning capability of LLMs while anchoring its outputs in both visual evidence and prior expert knowledge, significantly reducing hallucinations and improving clinical fidelity. A summary of models that use LLMs/VLMs can be seen in Table 2. 

### 2.3. Graph-Based Methods

One of the primary challenges in RRG research is bridging the gap between image and text modalities to generate clinically accurate reports. The use of graphs in RRG helps address this challenge by linking image and text data more effectively. Graph-based methods model the relationships between entities, such as anatomical structures, pathologies, and clinical findings, using nodes and edges. Two forms of graph representation in RRG are knowledge graphs and scene graphs.

Knowledge graphs incorporate prior medical knowledge, where nodes represent clinical concepts (e.g., “lung opacity” and “pneumothorax”) and edges encode semantic or causal relationships between them (e.g., “indicates” and “associated with”). These graphs can be used to guide the report generation process by constraining or informing the model based on established medical ontologies or diagnosis hierarchies.

Scene graphs, in contrast, are derived directly from visual data and capture the spatial or contextual relationships within an image. For radiology, this may include representations like “nodule inside lung” or “consolidation adjacent to pleura.” Scene graphs provide a structured abstraction of the visual content, allowing the model to reason over object interactions and generate more detailed and anatomically detailed descriptions.

#### 2.3.1. Knowledge Graphs

One of the most prominent tools used for RRG is knowledge graphs, which were formally introduced by Google in 2012 to enhance their search engine. Since then, knowledge graphs have played a significant role in various studies related to RRG.

A knowledge graph is a structured representation of knowledge where nodes represent entities or concepts, and edges define the semantic relationships between these entities or concepts. In some cases, entities also contain attributes [49]. In the context of RRG, knowledge graphs have been constructed around disease-related topics, with nodes representing disease labels and edges illustrating the relationships between them. X-ray images are used to extract visual features, which are then combined with the knowledge graph to generate RRGs [49]. Knowledge graphs are typically extracted from textual data, such as radiology reports [49,50,51,52,53], although some studies have attempted to derive them directly from radiologic images. Additionally, some approaches integrated text and image features to refine the knowledge graph’s nodes, linking regional image features with disease class embeddings extracted from the reports [42].

Knowledge graphs improve the accuracy and reliability of RRG models, particularly when combined with advanced modalities such as transformers [54], VLMs, and LLMs [42,49,52,55]. They are often employed as backbones for encoders and decoders. Gune et al. introduced the Knowledge Graph Augmented Vision Language BART (KGVL-BART), which takes two images as input, one frontal and one lateral, along with tags [52]. They also constructed a knowledge graph called chestX-KG from text reports in the IU X-ray dataset, which was verified by two experienced radiologists.

Knowledge graphs were used as knowledge bases to enhance the accuracy of generated reports [49,54,55]. Given the vast amount of specialized knowledge in the medical domain, structuring this knowledge into graphs makes it easier to integrate into modeling frameworks [56], improving understanding and data correlation between text and image modalities [50,55,57]. Additionally, knowledge graphs serve as repositories for prior knowledge to support RRG [49,54].

Hou et al. [55] developed a module called Knowledge Enhanced Attention (KEA) to mitigate bias in textual data by integrating the IU Medical Knowledge Graph (IU-MKG) [58]. This module mines relationships among medical findings and provides background medical information, combining this with the visual features extracted from radiological data [55]. Similarly, Kang et al. utilized knowledge graphs to bridge the gap between visual and textual data. They proposed a cross-modal knowledge-driven network (CKNet) to improve cross-modal knowledge transfer [57].

Another issue that knowledge graphs help alleviate is the lack of consideration for interconnections between diseases and symptoms, as well as between different diseases, in RRG [50,53]. Knowledge graphs address this by representing diseases and their associated symptom features, which are then combined with visual features extracted from images to generate radiology report [50,53].

As defined by Yin et al., contrastive learning is a self-supervised learning method often used with knowledge graphs to distinguish between similar and dissimilar sample pairs, enhancing the ability to extract data features [56]. It is employed to detect abnormal areas in radiologic images and to manage unlabeled medical images, which are common in the medical field. This method is particularly effective when dealing with unlabeled data, such as in radiology, and when multimodal alignment is required [55].

Given the excellent results achieved by knowledge graphs in RRG, the research focus has shifted toward improving their detail and scope [51]. Earlier methods used knowledge graphs that covered only a limited number of diseases and focused on those explicitly mentioned in reports, often neglecting normal or abnormal attributes associated with diseases, an oversight critical for medical accuracy in RRG [51,53]. To address this, one study proposed a novel approach called Divide-and-Conquer (DCG), which improves knowledge graphs by creating disease-free and disease-specific nodes. These nodes were extracted from reports, continuously distinguishing between normal conditions and specific diseases [51].

Zhang et al. created the ReXKG system to evaluate the constructed radiology knowledge graphs [59]. The system consists of three metrics: ReXKG-NSC to evaluate the similarity of nodes, REXKG-AMS to evaluate the distribution of edges, and REXKG-SCS to measure the coverage of sub-graphs across various knowledge graphs. They also conducted an in-depth analysis to comparing AI-generated reports with human-written reports. Their findings indicated that while generalist models trained on multiple modalities provided broader coverage and demonstrated improved radiological knowledge, they lacked the depth and detail typically found in human-written reports.

#### 2.3.2. Scene Graphs

In the field of CV, accurately interpreting and understanding visual scenes is critical for various applications, including manufacturing, augmented reality, and medical imaging. As these applications grew in complexity, there was a corresponding need for effective methods to handle the diverse and complex data involved. Additionally, the integration of NLP with CV helped advance CV applications and propel more research into applications such as image captioning, visual question answering (VQA), and visual dialog. These applications required a deeper and more accurate understanding of visual scenes [60]. Consequently, scene graphs, a visually grounded graph over the object attributes in a given image, was one of the methods introduced by Johnson et al. [61]. In the context of RRG, the graph nodes represent the object-bounding boxes with their object categories, while the edges depict their pair-wise relationships.

Scene graph generation (SGG) aims to localize and identify objects in images while visualizing their relationships. It serves as an auxiliary representation to improve image retrieval. The varied semantic concepts and compositional structures of visual relations resulted in a wide concept space, making this task complex. Visual scene graphs often include knowledge of the objects present, their associated properties, and the pairwise relationships between distinct objects, capturing the high-level semantic content of an image [62]. SGG has played an important role in enhancing and understanding the visual content in images.

SGG, as explained in [63], is a technique used to understand and visualize image content in a structured manner. The process involves first detecting and classifying objects, identifying their locations, and determining their types. Scene graphs have proved to be efficient in various CV and NLP applications, motivating researchers to integrate them into medical imaging applications such as RRG. However, only a few studies have explored the use of scene graphs for RRG, with two primary studies published in 2024.

First, Wang et al. introduced the Scene Graph-aided RRG (SGRRG) network [64]. SGRRG is a framework designed to achieve medical knowledge distillation in an end-to-end manner by generating region-level visual features, predicting anatomical attributes, and leveraging an automatically generated scene graph. Afterward, the scene graph is translated using a scene graph encoder, and a fine-grained token-type embedding method is introduced to help solve the problem of overlapping anatomical regions in radiology scene graphs. The encoded scene graph, along with the visual and textual tokens, were fed into a scene graph-aided decoder with a fine-grained distillation attention mechanism to extract the scene graph information into the model. By utilizing both global and local information, SGRRG improved its accuracy. SGRRG produced promising results on the MIMIC-CXR dataset, surpassing multiple state-of-the-art methods and showcasing superior capabilities in capturing abnormal findings.

**Figure 4:**
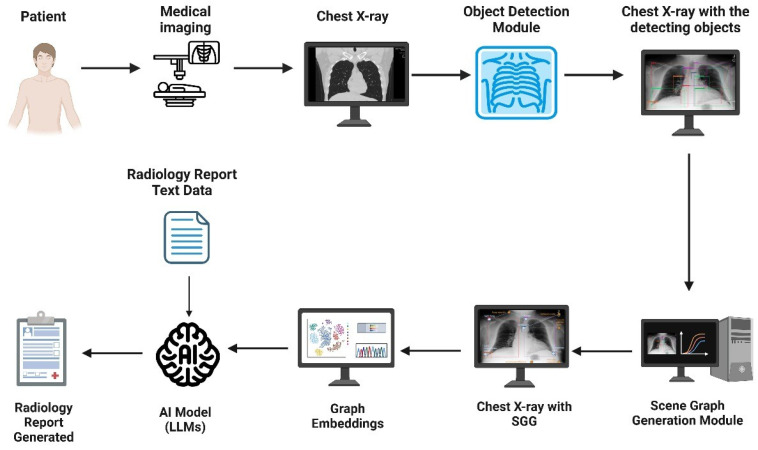
An example pipeline of scene graph generation (SGG) applied to radiology report generation (RRG). SGG represents one subclass of image-based RRG methods, and not a universal framework.

Second, Zhang et al. incorporated SGG into RRG due to traditional methods struggling to capture the significant semantic disparity between image and text modalities, which sometimes lead to suboptimal generated reports that may lack interpretability and does not take advantage of the spatial and contextual information present in the images [62]. Their framework utilizes a module named iterative scene graph generation, which aims to represent the relationships between anatomical regions in the image. This module also uses an auto-regressive scheme and a contextualization mechanism to enhance the scene graph. The enhanced scene graph, along with the learned attributes, is then used to generate the radiology reports, which are found to be accurate and interpretable, as they closely reflect the anatomical regions and relationships identified in the X-ray images. Their approach demonstrated exceptional results on the Chest ImaGenome dataset. Using these two papers, an overview of a representative scene graph pipeline for RRG is constructed in Figure 4. It is important to note that SGG represents only one subclass of image-based radiology report generation methods, and not a universal or standard approach across all models.

Additionally, scene graphs have expanded to other medical areas, such as the operating room, where semantic scene graphs were employed to model relationships between actors, including medical staff, patients, and equipment. These models enabled a better understanding of complex surgical images and functioned automatically, proving effective in tasks such as function prediction [65]. The development of medical semantic scene graphs (MSSGs) successfully provided a unified, symbolic, and spatial representation of surgical procedures. MSSGs modeled the relationships between objects, such as medical personnel, equipment, and imaging systems, highlighting the versatility and power of scene graphs in medical environments [66]. Scene graphs significantly impact medical accuracy and efficiency by establishing sequential relationships within medical data. They facilitate better data interpretation, improved surgical planning, enhanced decision-making in the operating room, and ultimately contribute to better patient outcomes. A brief summary of Graph-based models can be seen in Table 3.

## 3. Datasets

**Figure 5:**
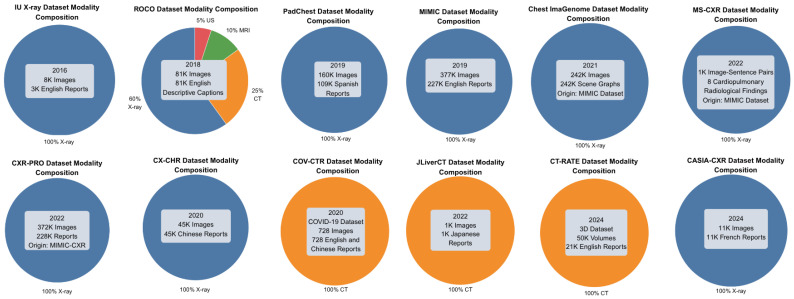
The dataset modality composition. Blue indicates X-ray, orange indicates CT scans, and green indicates MRI.

Medical imaging data, along with other forms of medical information, is often scarce and typically requires specific credentials for access. This limitation stems from the sensitive nature of the data and the ethical and confidentiality concerns associated with it. Consequently, many databases used for generating radiology reports are restricted and require accreditation for research purposes. Previous studies often sourced confidential data from unidentified or private clinics and hospitals. Even when some datasets are accessible, it remains essential for research to continue generating new datasets to ensure the availability of more organized and up-to-date information. Another limitation arises from the heterogeneity in dataset modalities. As shown in Figure 5, the majority of samples are X-rays, with only a small fraction being CT scans. Notably, the ROCO (RadiologyObjectsinCOntextdataset) dataset [67] includes a mix of modalities, though X-rays still dominate. This imbalance may introduce bias and limit the generalizability of the models’ findings across different imaging techniques.

For the purpose of RRG, we reviewed only those datasets that pair X-ray images with corresponding reports or descriptive captions. A summary of these datasets is provided in Table 4.

The Indiana University Chest X-ray Dataset (IU X-ray), introduced by Demner-Fushman et al. in 2016, consisted of 3996 radiology reports paired with 8121 associated images [68]. These were collected from the Indiana Network for Patient Care archive.

Radiology Objects in COntext (ROCO), presented by Pelka et al. in 2018 [67]. This multimodal image dataset contains over 81,000 images with various medical imaging modalities, including X-rays and CT, and their corresponding descriptive captions, all of which are retrieved from the open-access database PubMedCentral.

Pathology Detection in Chest Radiographs (PadChest), developed by Bustos et al. in 2019 [69]. It consists of 109,931 radiology reports in Spanish and 160,868 images obtained from 67,625 patients from the San Juan Hospital in Spain.

Medical Information Mart for Intensive Care Chest X-ray (MIMIC-CXR), introduced by Johnson et al. in 2019 [70], includes 377,110 images and 227,835 radiology reports from 65,379 patients seen in an emergency department between 2011 and 2016. MIMIC-CXR has served as the foundation for several derivative datasets, with the most notable being the following:

- MIMIC-CXR-JPG, by Johnson et al. in 2019 [71], provides the same data as MIMIC-CXR but in JPG format. Structured labels are automatically extracted from the corresponding free-text reports.
- Chest ImaGenome, by Wu et al. in 2021 [74], contains 242,072 images from the MIMIC-CXR dataset with automatically generated scene graphs. Each scene graph depicts a single frontal chest X-ray image and, when available in the corresponding radiology report, included phrases explaining 29 distinct anatomical regions of the chest, along with bounding box coordinates for those regions.
- MS-CXR, by Boecking et al. in 2022 [76], consists of 1162 image-sentence pairs that include bounding boxes and corresponding phrases. The dataset covers eight cardiopulmonary radiological findings, with an approximately equal number of pairs for each finding.
- CXR-PRO, by Ramesh et al. in 2022 [77], includes 371,920 images and 227,943 reports. This dataset extends MIMIC-CXR by removing references to prior radiological studies from reports using a BERT-based token classifier.

In addition, CX-CHR, introduced by Wang, Fuyu et al. in 2020 [72], is a private dataset containing 45,598 chest X-ray images and their corresponding reports in Chinese, collected from 35,609 patients. COV-CTR, developed by Li et al. in 2020 [73], is a COVID-19 CT dataset comprising 728 images with their corresponding reports available in both English and Chinese. JLiverCT, presented by Nishino et al. in 2022 [75], is a private dataset consisting of 1083 samples, with their reports in Japanese. CT-RATE, published by Hamamci et al. in 2024 [78], is a public 3D CT chest dataset containing 25,692 non-contrast chest CT volumes, later expanded to 50,188 volumes through various reconstructions. The dataset includes data from 21,304 unique patients, accompanied by their corresponding English radiology reports. CASIA-CXR, introduced by Metmer and Yang in 2024 [79], is a chest X-ray dataset containing 11,111 images and their corresponding radiology reports written in French. The data was collected from 11,111 patients across various hospitals in France.

## 4. Evaluation Metrics

After generating a radiology report, it is essential to ensure its accuracy and reliability. To achieve this, various evaluation metrics have been developed to assess the overall quality of the generated report, which is critical for evaluating the model’s performance. The generated reports must be both grammatically correct and factually accurate in terms of medical information. Typically, the evaluation involves comparing the AI-generated report with a radiologist-written report. Evaluation metrics are generally categorized into two types: quantitative and qualitative. Quantitative metrics assess the text quality and medical accuracy of the generated report, utilizing Natural Language Generation (NLG) and medical correctness metrics, respectively. On the other hand, qualitative evaluations are conducted by human experts, such as radiologists, who provide an overall assessment of the generated report. This section is organized into two subsections: metrics for text summarization, which focus on linguistic quality and coherence, and metrics for image-to-text generation, which emphasize clinical accuracy and alignment between images and text. An overview of these metrics can be seen in Table 5.

### 4.1. Evaluation Metrics for Text Summarization

These metrics are designed to evaluate the linguistic quality and coherence of generated radiology reports by comparing them to reference texts. Rooted in natural language processing (NLP), these metrics assess grammatical correctness, fluency, and semantic similarity, making them suitable for evaluating the textual output of RRG models. They ensure that generated reports are coherent and align with the structure and style of radiologist-written reports. The following are the most commonly used metrics in RRG as identified in the reviewed literature:

- BiLingual Evaluation Understudy (BLEU) [80]: BLEU measures the precision of position-independent n-grams, up to a length of four, between the generated and reference texts. Originally designed for machine translation, it evaluates uni-grams (BLEU-1), bi-grams, and so on, with scores closer to 1 indicating better performance [80]. BLEU is well-suited for RRG because it quantifies the overlap of word sequences, ensuring that generated reports closely mimic the structure and phrasing of expert-written reports, which is critical for maintaining clinical readability [80].
- Recall-Oriented Understudy for Gisting Evaluation (ROUGE) [81]: ROUGE, particularly ROUGE-L, is widely used in RRG for summarization tasks. It measures the length of the longest common subsequence between two texts, emphasizing recall in the weighted mean of precision and recall, and accounts for in-sequence matches to reflect sentence-level word order [81]. ROUGE is appropriate for RRG, as it evaluates how well the generated report captures the key content of the reference report, ensuring comprehensive coverage of the essential findings.
- Metric for Evaluation of Translation with Explicit ORdering (METEOR) [82]: METEOR extends BLEU-1 by incorporating both precision and recall, matching uni-grams based on exact form, stemmed form, or meaning. It uses the harmonic mean of uni-gram precision and recall, with a bias toward recall, and rewards identically ordered uni-grams [82]. METEOR is valuable in RRG for its ability to account for synonyms and semantic equivalence, ensuring that generated reports convey the same clinical intent as reference reports, even if worded differently.
- BERTScore [83]: BERTScore calculates similarity scores for each token in the generated text against tokens in the reference text using contextual embeddings [83]. It is well-suited for RRG because it captures semantic equivalence beyond exact word matches, allowing for a more nuanced evaluation of reports that may use varied but clinically equivalent terminology.

These metrics are critical for assessing the linguistic quality of RRG outputs, ensuring that reports are not only accurate but also coherent and professionally structured, which is essential for clinical usability.

### 4.2. Evaluation Metrics for Image-to-Text Generation

These metrics are designed to measure the clinical accuracy and alignment between radiological images and the generated reports. They evaluate how well the model interprets visual data and translates it into diagnostically relevant text, a core requirement for RRG applications. These metrics are particularly suited for assessing the clinical validity of reports, ensuring that abnormalities and findings are correctly identified and described. The following metrics are commonly used:

- Consensus-based Image Description Evaluation (CIDEr) [84]: CIDEr measures the cosine similarity between Term Frequency-Inverse Document Frequency (TF-IDF) weighted n-grams, focusing on content words and applying a Gaussian penalty to reward length similarity [84]. Originally developed for image captioning, CIDEr is effective in RRG because it evaluates how well the generated report captures the key visual content of the radiological image, emphasizing clinically relevant terms and findings.
- Clinical Efficacy (CE) [85]: CE, introduced by Chen et al., utilizes the CheXpert labeler to generate 14 labels for thoracic pathologies and support devices in both generated and reference reports [85,86]. It is tailored for the MIMIC-CXR dataset due to CheXpert’s specific labeling schema. A high CE score indicates substantial semantic similarity between the generated and reference reports. CE is crucial for RRG, as it directly measures the clinical accuracy of identified pathologies, ensuring that the model correctly interprets medical images.
- Semb [87]: Proposed by Endo et al., Semb uses CheXpert to evaluate semantic equivalence between generated and reference reports through cosine similarity [87]. This metric is effective for RRG because it assesses whether the generated report conveys the same clinical meaning as the reference, even if the phrasing differs, which is vital for diagnostic reliability.
- Radiology Report Quality Index (RadRQI) [88]: RadRQI, introduced by Yan et al., evaluates the accuracy of radiology-related abnormalities and their clinical attributes using RadLex for keyword extraction and RadGraph for contextualizing relationships, including negations [88,89,90]. The RadRQI-F1 score reflects the correctness of abnormalities and their attributes. RadRQI is well-suited for RRG, as it ensures that the generated report accurately describes clinical findings and their relationships, aligning with the diagnostic intent of the image.
- MRScore [91]: Liu et al. introduced MRScore, which leverages Reinforcement Learning with Human Feedback (RLHF) to train a pre-trained LLM (Mistral-7B-instruct) to assess report quality [91]. Using a dataset of high- and lower-quality report pairs from MIMIC-CXR, radiologists assigned quality scores (90, 60, or 30). MRScore assigns higher values to higher-quality reports, aligning with expert preferences. This metric is particularly valuable for RRG, as it integrates human expertise to evaluate both clinical accuracy and overall report quality, addressing the nuanced requirements of medical reporting [91].
- RadCliQ-v1 [12]: RadCliQ-v1, a composite metric used by the MedVersa model, integrates traditional and domain-specific measures to evaluate both linguistic quality and clinical accuracy [12]. It achieved a score of 1.46 ± 0.03 on the IU X-ray findings sections, outperforming other AI systems in clinical relevance [12]. RadCliQ-v1 is ideal for RRG because it provides a comprehensive assessment, balancing text quality with clinical fidelity, ensuring that reports are both readable and diagnostically sound.

These metrics are essential for validating the clinical relevance of RRG models, ensuring that the generated reports accurately reflect the visual information in radiological images and support clinical decision-making. For example, Zhang et al. (2021) demonstrated strong performance in clinical accuracy using metrics like RadGraph F1, highlighting the importance of contextual relationships in report evaluation [49].

### 4.3. Human-Based Metrics

Given the complexity of human language, particularly in medical texts like radiology reports, evaluating their performance remains challenging. For this reason, some studies opted for qualitative assessments by human experts. While evaluations by clinical specialists are considered the most reliable, they are both expensive and time-consuming. Asking overburdened healthcare professionals to review generated reports adds to their workload. An important area of research focuses on developing appropriate methods to assess the medical semantics and fluency of generated reports.

## 5. Comparative Analysis and Discussion

This section reviews the results of the top-performing models by comparing their reported metrics: BLEU, ROUGE, METEOR, and CE. All recorded values were extracted from the original papers, with evaluations conducted on the IU X-ray [68] and MIMIC-CXR [70] datasets. Table 6 highlights the top-performing model on the MIMIC-CXR dataset, which is the RADAR model developed by Hou et al. [48]. This model focuses on improving report generation by leveraging both the internal knowledge of an LLM and externally retrieved information. Their results demonstrate superior performance across nearly all key evaluation metrics, including the highest scores in BLEU-1 (0.509), BLEU-4 (0.262), ROUGE (0.397), and METEOR (0.450), indicating its strong capability in both linguistic quality and clinical relevance. The results supported their claim that integrating internal LLM knowledge with external domain-specific retrieval allows it to generate more accurate and context-aware reports. The AP-ISG model by Zhang et al. achieved the second-highest scores. AP-ISG is a novel region-based Attribute Prototype-guided Iterative Scene Graph (AP-ISG) framework for report generation [62]. It utilizes SSG as an auxiliary task, enhancing interpretability and relational reasoning capabilities.

**Figure 6:**
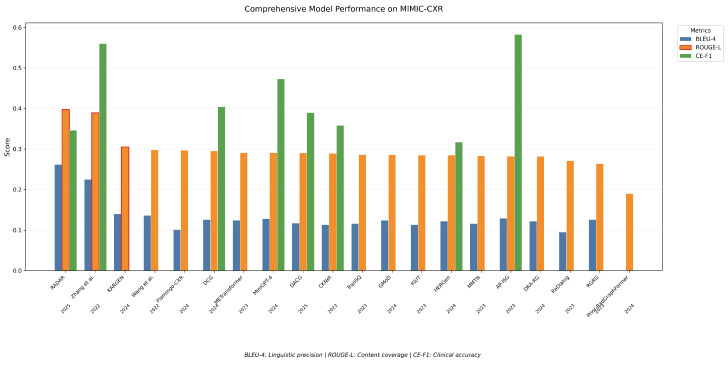
Models’ performance for BLEU-4, ROUGE, and CE-F1 metrics on the MIMIC-CXR dataset.

Figure 6 further supports this by visualizing the scores on the three metrics BLEU-4, ROUGE, and CE-F1. While the AP-ISG model [62] shows the highest CE-F1 (0.582), indicating superior performance in classification-related tasks such as abnormality identification, it shows relatively average scores for both BLEU-4 (0.129) and ROUGE (0.282), suggesting its outputs may lack linguistic fluency or the comprehensive coverage of clinical findings compared to other models. In contrast, Zhang et al. [49] achieved higher balanced scores across all three metrics (e.g., BLEU-4: 0.225, ROUGE: 0.389, and CE-F1: 0.56), demonstrating better performance in both language generation and clinical accuracy. This aligns with their emphasis on integrating prior knowledge graphs, which likely improved both semantic relevance (reflected in ROUGE) and term precision (BLEU-4), while maintaining competitive CE-F1 for task-specific classification.

Table 7 presents the top-performing models on the IU X-ray dataset, which include the IIHT model by Fan et al. [37] and the model developed by Tsaniya et al. [35]. Both models are transformer-based, reinforcing the idea that transformer architectures serve as an effective foundation for RRG.

**Figure 7:**
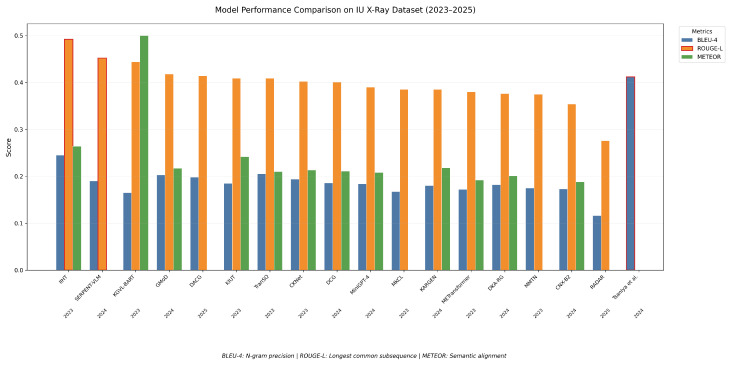
Models’ performance for BLEU-4, ROUGE, and METEOR metrics on the IU X-ray dataset.

However, as seen in Figure 7, while IIHT [37] performs well on the ROUGE score (0.492), indicating strong recall in capturing key clinical concepts from the reference reports, and Tsaniya et al. [35] excel in BLEU-4 (0.412), reflecting higher precision in term usage, the KGVL-BART model [52] demonstrates a distinct trade-off. It achieves competitive performance on both ROUGE (0.444) and METEOR (0.5), suggesting robust semantic alignment with reference texts and the balanced coverage of salient content. Yet, its notably low BLEU-4 score (0.165) implies limitations in lexical overlap with ground-truth reports.

Overall, the analysis of results from the two mainstream datasets for RRG indicates that several architectures have achieved exceptional performance. The integration of knowledge graphs and transformers has been consistently reported as a key factor in improving the accuracy and quality of RRG.

## 6. Open Issues

### 6.1. Generalization

In the domain of RRG, one of the most significant challenges is the limitation of generalization. Due to the nature of medical datasets, most models are trained on specific datasets or formats, which makes generalization difficult [38,40]. This reliance on single-source datasets introduces a risk of bias, potentially hindering model performance when applied to radiology reports from other settings [42,44]. Additionally, the quality and variability of input data play a critical role in determining a model’s ability to generalize, further complicating its application in diverse clinical contexts [38,39].

### 6.2. Computational Expense

Another common issue is the significant computational resources required for fine-tuning and inference, which may limit adoption by smaller research teams or institutions [14]. This poses scalability challenges for real-time clinical applications [40], particularly when expanding to more complex medical conditions or adapting to the continuously evolving knowledge landscape in medicine [42].

### 6.3. Complexity

RRG is inherently complex due to the intricacy of human language in radiology reports and the hidden features in medical images. As a result, some models struggle with complex cases requiring a deeper understanding or with conditions underrepresented in the training data [14]. For instance, while KARGEN effectively fuses visual and textual features, there remains room for improvement in its understanding of complex disease relationships and medical terminology [42].

### 6.4. Limited Views

Another issue is the limitation of many models to single X-ray images, which prevents them from processing multi-view or longitudinal imaging data, essential elements for comprehensive diagnostics [39]. Additionally, model performance may degrade when applied to other medical images or modalities [40].

### 6.5. The Black-Box Challenge

Generative models face significant limitations due to their black-box nature, which obscures how decisions are made and hinders clinical interpretability. Unlike simpler, rule-based systems, these complex models produce outputs that clinicians cannot easily explain, slowing their adoption in real-world settings. Because the decision-making process is not transparent, it becomes difficult to detect when the model makes critical errors, for instance, hallucinating rare diseases when such conditions are underrepresented in the training data. This lack of insight means biases often go unnoticed and unaddressed. Post hoc methods like RadFlag attempt to identify uncertain predictions by analyzing the outputs, but they do not explain the root causes of errors or suggest corrective strategies. Moreover, these methods rely on threshold settings specific to each classifier, which limits their applicability across diverse tasks. Compounding the issue, black-box models often exhibit inconsistent performance across different classes of findings, indicating potential robustness concerns. Improving the reliability of these systems, and building clinicians’ trust, will require the development of interpretable models that can provide meaningful explanations for their decisions [40].

### 6.6. Model Hallucinations

One of the most critical issues in LLMs or VLMs is their tendency to hallucinate, generating erroneous details such as incorrect dates or comparisons to non-existent prior reports [41,45]. This issue poses significant challenges in ensuring the reliability and accuracy of generated outputs.

### 6.7. Lack of Fine-Grained Error Analysis

It is a weakness in the current evaluation methods for LLM radiology reports that no subtle mistakes are analyzed. Most such evaluation metrics, such as BLEU and ROUGE, look at the general text similarity but still miss out on important clinical errors like missed diagnoses and false descriptions. It is confusing to know which problems with Al can be fixed without close examination and testing by clinicians. It is therefore necessary to establish uniform guidelines that use the knowledge of medical experts to look for errors in each part of the report to advance the reliability and safety of machine-created reports [92].

## 7. Future Research Directions

### 7.1. Advanced Knowledge Integration and Utilization

Future RRG systems should go beyond static knowledge graphs by incorporating real-time updates. These graphs could integrate the latest medical research findings and patient-specific data, ensuring that systems remain current and adaptable to diverse clinical scenarios. For example, dynamic graph systems could prioritize emergent diseases like COVID-19 during pandemics, enhancing their responsiveness to global health crises. Additionally, while current systems often limit nodes to diseases or anatomical regions, expanding these to include nuanced relationships, such as temporal disease progression and multi-organ impacts, could significantly improve diagnostic depth.

### 7.2. Mitigating Hallucination and Ensuring Trustworthiness

Hallucination remains a critical challenge in RRG. Future research should focus on Reinforcement Learning with Human Feedback (RLHF) to align generated outputs with expert-validated standards. Techniques like entailment-based evaluation, as demonstrated by RadFlag [40], show promise in detecting and reducing errors. Incorporating human oversight during both model training and deployment phases can further ensure that the generated reports align with clinical realities. Interactive validation by radiologists would enhance safety and reliability, particularly in critical cases.

### 7.3. Expansion of Multimodal and Longitudinal Data Usage

While most RRG systems primarily focus on chest X-rays or CT scans, expanding their capabilities to include PET scans, ultrasounds, and functional imaging would greatly enhance versatility. Such an expansion would allow systems to cater to specialties like oncology and cardiology, where multimodal imaging is critical. Models like MAIRA [43] have demonstrated the benefits of leveraging multiple views for richer diagnostic insights. Incorporating longitudinal data, as seen in HERGen [44], could contextualize findings by analyzing disease progression, treatment efficacy, and patient-specific trends, significantly advancing personalized medicine.

### 7.4. Ethical, Regulatory, and Interpretability Challenges

Interpretability is essential for clinical adoption. Future RRG systems should prioritize models that explain their decision-making processes, such as graph-based models (e.g., scene graphs). These systems could offer clear mappings between input data and outputs, enabling radiologists to efficiently verify findings. Future research must also address biases inherent in datasets and training processes. Models should be evaluated for their performance across diverse patient demographics, imaging equipment, and healthcare systems to ensure equitable access and accuracy. Collaboration with regulatory bodies will be crucial to establish guidelines for deploying AI systems in healthcare, including rigorous validation protocols to ensure safety and reliability.

### 7.5. Universal Evaluation Metrics

Current evaluation metrics often lack universality, being tied to specific datasets like MIMIC-CXR. Future efforts should focus on developing dataset-agnostic benchmarks, enabling fair comparisons across models and datasets. Metrics like MRScore [91], which align model outputs with expert judgments, should be refined and adopted as standard tools.

### 7.6. Dataset Expansion and Accessibility

Most existing datasets are English-centric, limiting their utility in non-English-speaking regions. Future initiatives should prioritize creating annotated datasets in multiple languages to support global adoption of RRG systems. Datasets like CASIA-CXR [79], which provide French-language reports, are a promising step in this direction.

## 8. Conclusions

This study comprehensively examined the latest advancements in RRG from the years 2022 to 2024, encompassing key methods, prominent datasets, and widely used evaluation metrics. It highlighted the transformative role of advanced AI technologies, including transformers, VLMs, and LLMs in addressing the growing demand for radiological services and alleviating the workload of radiologists. These innovations have demonstrated significant improvements in diagnostic accuracy, report quality, and overall efficiency in clinical workflows. The review highlighted the effect of integrating domain-specific knowledge bases, such as knowledge graphs and scene graphs, into RRG frameworks. These tools have proven effective in enhancing the accuracy, interpretability, and context-awareness of generated reports. Additionally, robust architectures, such as transformers and hybrid models that combine CNNs with transformers, consistently outperformed traditional methods. Despite these advancements, challenges persist. Hallucinations, semantic inaccuracies, and domain adaptation issues remain critical obstacles that need to be addressed to ensure the reliability and trustworthiness of RRG systems. Furthermore, most models face limitations in generalizability due to their reliance on specific datasets or imaging modalities, highlighting the need for frameworks capable of handling multimodal and longitudinal data. The growing availability of tailored datasets, particularly those for chest X-rays and CT scans, has played a pivotal role in advancing the RRG domain. However, the field remains constrained by the scarcity of publicly accessible and multi-language datasets, limiting its global applicability. Future efforts should prioritize creating and sharing diverse, annotated datasets across languages and imaging modalities to support the development of more inclusive and generalizable RRG systems. Finally, the study emphasized the importance of expanding evaluation metrics beyond traditional text quality measures to include semantic accuracy, clinical relevance, and medical correctness. Metrics such as MRScore and Clinical Efficacy align AI-generated outputs with expert human evaluations, bridging the gap between machine-generated content and clinical needs. Collaborative efforts between researchers, and clinicians will be crucial in overcoming existing challenges and driving the development of trustworthy, effective, and globally adaptable RRG systems.

## Figures and Tables

**Table 1:** Summary of RRG using deep learning methods. All models are applied using chest X-ray radiographs.

Model	Architecture	Data	Advantages	Limitations
METransformer [20]	Transformer	IU-Xray, MIMIC-CXR	Uses expert tokens to focus on diverse image regions for detailed diagnostics.	Basic framework without integrated external medical knowledge.
Wang et al. [21]	Transformer	MIMIC-CXR	Memory-augmented sparse attention captures high-order clinical context.	Attention mechanisms capture only first-order interactions, missing complex feature relationships.
KiUT [22]	Transformer	IU-Xray, MIMIC-CXR	Combines visual, contextual, and graph-based clinical knowledge.	Relies on expert-constructed symptom graphs.
CT2Rep [23]	Transformer	CT-RATE	Specialized for 3D CT data with dual transformers and relational memory.	No directly comparable prior methods; evaluation is limited to internal baselines.
GIT-CXR [24]	Transformer	MIMIC-CXR	Curriculum learning enables robust generation of long, detailed reports.	Curriculum learning is based only on report length, not tailored specifically to medical imaging.
PPKED [26]	CNN + Transformer	IU-Xray, MIMIC-CXR	Merges prior and posterior knowledge via attention for contextual accuracy.	Multiple modules increase model complexity and computational cost.
CNX-B2 [27]	CNN + Transformer	IU-Xray	Combines ConvNeXt with BioBERT for strong visual-semantic alignment.	High computational complexity and large parameter count hinder deployment on low-resource devices.
TranSQ [28]	CNN + Transformer	IU-Xray, MIMIC-CXR	Simulates radiologist workflow via semantic queries and attention.	Limited linguistic flexibility due to sentence retrieval instead of free-form generation.
MMTN [29]	CNN + Transformer	IU-Xray, MIMIC-CXR	Memory-based attention aligns key visual cues with disease terminology.	High computational complexity limits deployment on low-resource devices.
RGRG [30]	CNN + Transformer	Chest ImaGenome	Region-aware detection highlights anatomical and pathological zones.	Fails to generate image-level findings not tied to anatomical regions.
DACG [32]	CNN + Transformer	IU-Xray, MIMIC-CXR	Context guidance memory improves long-form, coherent generation.	Needs more detailed and structured updates to the guidance memory.
Liu et al. [34]	CNN + LSTM + Transformer	IU-Xray, MIMIC-CXR	Highlights abnormalities via contrast with normality pool.	Contrastive attention is applied only on global features, limiting local detail sensitivity.
Zhou et al. [35]	CNN + LSTM + Transformer	IU-Xray, MIMIC-CXR	BioSentVec encodes patient context for diagnostic consistency.	Template-driven semantic module restricts report diversity and expressiveness.
Tsaniya et al. [37]	CNN + LSTM + Transformer	IU-Xray	BERT + LSTM fusion ensures precise and coherent term usage.	Diverse reports for similar conditions affect model convergence during training.
IIHT [38]	CNN + LSTM + Transformer	IU-Xray	Conditions report generation on disease indicators and structured reasoning.	Model performance is affected by accessibility and accuracy of disease indicator information.

**Table 2:** Summary of RRG using LLMs/VLMs.

Model	Modality	Anatomical District	Architecture	Data	Advantages	Limitations
Voinea et al. [38]	MRI, CT	10 regions	LLaMA 3-8B	University of Medicine and Pharmacy of Craiova’s Imaging Center	Tailors a general LLM to radiology using domain-specific fine-tuning.	Struggles with ambiguous or borderline cases, risking overconfident or overly cautious conclusions.
RaDialog [39]	Chest X-ray	Chest	BioViL-T + CLIP + Vicuna-7B	MIMIC-CXR, RaDialog-Instruct, IU-Xray	Ensures clinical accuracy using a CheXpert-based pathology classifier.	Best suited for simpler cases; not yet reliable for complex diagnostic scenarios.
Liu et al. [14]	Chest X-ray	Chest	MiniGPT-4	IU-Xray, MIMIC-CXR	Achieves high precision by filtering errors and irrelevant content.	Multi-stage architecture increases computational complexity, affecting deployment in low-resource environments.
RadFlag [40]	Chest X-ray	Chest	GPT-4	MIMIC	Detects hallucinations in generated reports via entailment validation.	Hallucination detection lacks extensive clinical validation beyond limited clinician agreement.
Udomlapsakul et al. [41]	Chest X-ray	Chest	SigLIP + Phi-2	MIMIC-CXR, LLaVa-Med 500k, CheXpert, PadChest, BIMCV COVID-19	Combines domain-specific pre-training and fine-tuning with safety prompts.	Sequential two-stage post-processing may introduce complexity and latency.
KARGEN [42]	Chest X-ray	Chest	Swin Transformer + LLaMA2-7B	MIMIC-CXR, IU-Xray	Combines vision features and medical knowledge via GCN and Swin Transformer.	Knowledge graph is limited to 14 CheXpert disease terms, reducing generalizability to less common conditions.
MAIRA [43]	Chest X-ray	Chest	MAIRA-1 + RAD-DINO + Vicuna + GPT-4	MIMIC-CXR, CheXpert, PadChest, BIMCV-COVID19, Open-I	Uses GPT-4 for candidate selection to minimize hallucinations.	May struggle with rare or uncommon medical conditions due to limited training representation.
HERGen [44]	Chest X-rays	Chest	DistilGPT2 + CXR-BERT	MIMIC-CXR	Tracks disease progression and reduces reporting inconsistencies.	May be less suitable for deployment in resource-constrained environments.
MiniGPT-Med [45]	Chest X-ray	Chest	MiniGPT-Med	MIMIC-CXR	Excels at both grounding and non-grounding radiology tasks.	Limited by the relatively small size of fine-tuning datasets, potentially reducing generalizability.
Flamingo-CXR [6]	Chest X-ray	Chest	Flamingo	MIMIC-CXR, IND1	Fine-tuned with radiologist feedback for clinical robustness.	Lacks access to contextual clinical data (e.g., lateral views, patient history), reducing diagnostic accuracy.
SERPENT-VLM [46]	Chest X-ray	Chest	SERPENT-VLM	IU-Xray, ROCO	Reduces hallucinations and boosts accuracy on noisy datasets.	May struggle with variable image quality in real-world applications.
iHealth-Chile-1 [47]	Chest X-ray	Chest	CLIP + BiomedCLIP + LLaMA + Vicuna	CheXpert	Template-guided prompts ensure structured and concise reporting.	May not fully match human-level natural language quality across all evaluation dimensions.
RADAR [48]	Chest X-ray	Chest	BLIP-3	MIMIC-CXR	Combines internal LLM reasoning with external knowledge.	Relies on external knowledge bases whose quality may affect report accuracy.

**Table 3:** Summary of RRG using graphs.

Model	Modality	Anatomical District	Architecture	Data	Advantages	Limitations
KGVL-BART [52]	Chest X-ray	Chest	Vision-Language BART with KG backbone	IU X-ray, MIMIC-CXR	Incorporates domain-specific knowledge; uses dual views (frontal/lateral); radiologist-verified KG	Relies on quality of KG construction; limited to specific dataset
KEA with IU-MKG [55]	Chest X-ray	Chest	Knowledge Enhanced Attention Module	IU-RR, CheXpert, ChestX-ray8	Reduces bias in textual data; enhances visual-text integration	Focused on known disease relations; scalability issues
CKNet [57]	Chest X-ray	Chest	Cross-modal knowledge-driven network	MIMIC-CXR, IU X-ray	Bridges modality gap; improves knowledge transfer	Limited exploration beyond single anatomical region
DCG-enhanced KG [51]	Chest X-ray	Chest	Divide-and-Conquer KG enhancement	MIMIC-CXR, IU X-ray	Distinguishes between normal and disease-specific findings	Complex KG generation pipeline
SGRRG [64]	Chest X-ray	Chest	Scene Graph Encoder + Decoder + Distillation Attention	MIMIC-CXR	Fine-grained visual-text alignment; solves overlapping region issue; SOTA performance	Requires accurate scene graph generation; complex architecture
Iterative SGG [62]	Chest X-ray	Chest	Iterative Scene Graph Generation + Auto-regressive Scheme	Chest ImaGenome	High interpretability; contextual representation of anatomical relations	Dependent on robustness of iterative module
MSSGs [66]	Image + Contextual Metadata	Surgery/OR scenes	Medical Semantic Scene Graph	Surgical imaging and metadata	Unified symbolic spatial representation; useful for surgical planning and actor interaction modeling	Applied primarily in surgical context; less focus on diagnostic report generation

**Table 4:** Available radiology datasets with radiology reports.

Name	Year	Avail.	Modality	Patients	Images	Reports	Language	Benchmark
IU X-ray [68]	2016	Public	X-ray	3955	8121	3996	English	✓
ROCO [67]	2018	Public	XR, CT, MRI, US	-	81,000	81,000	English	✓
PadChest [69]	2019	Restricted	X-ray	67,625	160,868	109,931	Spanish	✓
MIMIC-CXR [70]	2019	Restricted	X-ray	65,379	377,110	227,835	English	✓
MIMIC-CXR-JPG [71]	2019	Restricted	X-ray	65,379	377,110	227,835	English	✓
CX-CHR [72]	2020	Private	X-ray	35,609	45,598	45,598	Chinese	✓
COV-CTR [73]	2020	Public	CT	-	728	728	English, Chinese	✓
Chest ImaGenome [74]	2021	Restricted	X-ray	-	242,072	-	English	✓
JLiverCT [75]	2022	Private	CT	-	1083	1083	Japanese	✓
MS-CXR [76]	2022	Restricted	X-ray	-	1162	-	English	✓
CXR-PRO [77]	2022	Restricted	X-ray	65,379	371,920	227,943	English	✓
CT-RATE [78]	2024	Public	CT	21,304	50,188	25,692	English	–
CASIA-CXR [79]	2024	Public	X-ray	11,111	11,111	11,111	French	–

**Table 5:** Summary of evaluation metrics and their usage in RRG.

Metric Name	Usage
BLEU	Text summarization
ROUGE	
METEOR	
BERTScore	
CIDEr	Image-to-text generation
Clinical Efficacy (CE)	
Semb	
Radiology Report Quality Index (RadRQI)	
MRScore	

**Table 6:** Models’ performance on the MIMIC-CXR dataset. Bold indicates the highest metric achieved.

Year	Model	BLEU-1	BLEU-2	BLEU-3	BLEU-4	ROUGE	CIDEr	METEOR	CE-P	CE-R	CE-F1
2022	Zhang et al. [49]	0.491	**0.358**	**0.278**	0.225	0.389	-	0.215	**0.587**	0.593	0.56
	Wang et al. [21]	0.413	0.266	0.186	0.136	0.298	0.492	0.17	-	-	-
2023	RaDialog [39]	0.346	-	-	**0.95**	0.271	-	0.14	-	-	-
	AP-ISG [62]	0.391	0.258	0.182	0.129	0.282	**0.526**	0.175	0.518	**0.695**	**0.582**
	CKNet [57]	0.356	0.239	0.157	0.113	0.289	0.118	0.146	0.423	0.348	0.358
	METransformer [20]	0.386	0.25	0.169	0.124	0.291	0.362	0.152	-	-	-
	KiUT [22]	0.393	0.243	0.159	0.113	0.285	-	0.16	-	-	-
	RGRG [30]	0.373	0.249	0.175	0.126	0.264	0.495	0.168	-	-	-
	TranSQ [28]	0.423	0.261	0.171	0.116	0.286	-	0.168	-	-	-
	MMTN [29]	0.379	0.238	0.159	0.116	0.283	-	0.161	-	-	-
2024	MINIGPT-4 + I3 + C2FD [14]	0.402	0.262	0.18	0.128	0.291	-	0.175	0.465	0.482	0.473
	HERGen [44]	0.395	0.248	0.169	0.122	0.285	-	0.156	0.415	0.301	0.317
	KARGEN [42]	0.417	0.274	0.192	0.14	0.305	0.289	0.165	-	-	-
	Flamingo-CXR [6]	-	-	-	0.101	0.297	0.138	-	-	-	-
	GMoD [50]	0.398	0.251	0.172	0.124	0.286	0.377	0.166	-	-	-
	DCG [51]	0.397	0.258	0.166	0.126	0.295	0.445	0.162	0.441	0.414	0.404
	DKA-RG [56]	0.395	0.254	0.165	0.122	0.282	0.303	0.154	-	-	-
	Prior-RadGraphFormer [54]	0.07	-	-	-	0.19	-	0.07	-	-	-
2025	GIT-CXR (MV+C+CL) [24]	0.403	0.286	0.215	0.168	0.312	-	0.369	-	-	-
	GIT-CXR (SV+C+CL) [24]	0.393	0.278	0.208	0.162	0.305	-	0.359	-	-	-
	DACG [32]	0.398	0.249	0.167	0.117	0.290	-	0.162	0.422	0.405	0.389
	RADAR [48]	**0.509**	-	-	**0.262**	**0.397**	-	**0.450**	-	-	0.346

**Table 7:** Models’ performance on the IU X-ray dataset. Bold indicates the highest metric achieved.

Year	Model	BLEU-1	BLEU-2	BLEU-3	BLEU-4	ROUGE-L	CIDEr	METEOR
2023	MKCL [55]	0.49	0.311	0.222	0.167	0.385	0.523	-
	CKNet [57]	0.515	0.354	0.257	0.194	0.402	0.392	0.213
	KGVL-BART [52]	0.423	0.256	0.194	0.165	0.444	-	**0.5**
	METransformer [20]	0.483	0.322	0.228	0.172	0.38	0.435	0.192
	KiUT [22]	0.525	0.36	0.251	0.185	0.409	-	0.242
	TranSQ [28]	0.516	0.365	0.272	0.205	0.409	-	0.21
	MMTN [29]	0.486	0.321	0.232	0.175	0.375	0.361	-
	IIHT [37]	0.513	**0.375**	0.297	0.245	**0.492**	-	0.264
2024	MINIGPT-4 + I3+C2FD [14]	0.499	0.323	0.238	0.184	0.39	-	0.208
	SERPENT-VLM [46]	**0.547**	0.356	0.242	0.19	0.452	-	-
	KARGEN [42]	0.49	0.323	0.232	0.18	0.385	0.491	0.218
	GMoD [50]	0.53	0.363	0.267	0.203	0.418	0.437	0.217
	DCG [51]	0.514	0.33	0.241	0.186	0.401	0.578	0.211
	DKA-RG [56]	0.496	0.328	0.239	0.182	0.376	**0.621**	0.201
	CNX-B2 [27]	0.479	0.363	0.261	0.173	0.354	0.408	0.188
	Tsaniya et al. [35]	0.363	0.371	**0.388**	**0.412**	-	-	-
2025	DACG [32]	0.518	0.355	0.260	0.198	0.414	0.415	-
	RADAR [48]	-	-	-	0.116	0.276	-	-

## Abbreviations

AI	Artificial Intelligence
VLM	Vision Language Model
LLM	Large Language Model
RRG	Radiology Report Generation
CT	Computed Tomography
MRI	Magnetic Resonance Imaging
NLP	Natural Language Processing
ViT	Vision Transformer
CLIP	Contrastive Language–Image Pre-training
MSA	Memory-Augmented Sparse Attention
MCGN	Medical Concept Generation Network
KiUT	Knowledge-Injected U-Transformer
MBERT	Multilingual BERT
GAT	Graph Attention Network
MCLN	Memory-Driven Conditional Layer Normalization
PPKED	Posterior-and-Prior Knowledge Exploration and Distillation
PoKE	Posterior Knowledge Explorer
MKD	Multi-domain Knowledge Distiller
ADA	Adaptive Attention
CNN	Convolutional Neural Networks
AUROC	Area Under the Receiver Operating Characteristic
RNN	Recurrent Neural Networks
MCLN	Multiple Context Learning Networks
GIT	Generative Image-to-text Transformer
MMTN	Multimodal Memory Transformer Network
BERT	Bidirectional Encoder Representations
LSTM	Long short-term Memory
CLAHE	Contrast Limited Adaptive Histogram Equalization
EFF	Exposure Fusion Framework
IIHT	Image-to-Indicator Hierarchical Transformer
C2FD	Coarse to Fine Decoding
KARGEN	Knowledge-enhanced Automated Radiology Report Generation
GCN	Graph Convolutional Network
MIMIC-CXR	Medical Information Mart for Intensive Care Chest X-ray dataset
IU-Xray	Indiana University X-ray dataset
MAIRA	Multimodal AI for Radiology Applications
HERGen	History Enhanced Radiology Report Generation
ROCO	Radiology Objects in COntext dataset
BART	Bidirectional and Auto-Regressive Transformer
KGVL-BART	Knowledge Graph Augmented Vision Language BART
KEA	Knowledge Enhanced Attention
IU-MKG	IU Medical Knowledge Graph
CKNeT	Cross-Modal Knowledge-driven Network
DCG	Divide-and-Conquer
VQA	Visual Question Answering
SG	Scene Graph
SGG	Scene Graph Generation
MSSG	Medical Semantic Scene Graph
NLG	Natural Language Generation
BLEU	Bilingual Evaluation Understudy
ROUGE	Recall-Oriented Understudy for Gisting Evaluation
METEOR	Metric for Evaluation of Translation with Explicit Ordering
CIDEr	Consensus-based Image Description Evaluation
TF-IDF	the Term Frequency-Inverse Document Frequency
RadRQI	Radiology Report Quality Index
RLHF	Reinforcement Learning with Human Feedback
CXR	Chest X-ray

## References

[1] Rawson J.V., Smetherman D., Rubin E. (2024). Short-Term Strategies for augmenting the national Radiologist workforce. Am. J. Roentgenol..

[2] Liao Y., Liu H., Spasić I. (2023). Deep learning approaches to automatic radiology report generation: A systematic review. Inform. Med. Unlocked.

[3] Nishio M., Matsunaga T., Matsuo H., Nogami M., Kurata Y., Fujimoto K., Sugiyama O., Akashi T., Aoki S., Murakami T. (2024). Fully automatic summarization of radiology reports using natural language processing with large language models. Inform. Med. Unlocked.

[4] Miura Y., Zhang Y., Tsai E.B., Langlotz C.P., Jurafsky D. (2021). Improving Factual Completeness and Consistency of Image-to-Text Radiology Report Generation. arXiv.

[5] Aborizka M.A.M. Automated radiology report generation from chest X-ray images using CheXNet and Transformer-LSTM architecture. Proceedings of the IDDM’24: 7th International Conference on Informatics & Data-Driven Medicine.

[6] Tanno R., Barrett D.G.T., Sellergren A., Ghaisas S., Dathathri S., See A., Welbl J., Lau C., Tu T., Azizi S. (2025). Collaboration between clinicians and vision–language models in radiology report generation. Nat. Med..

[7] Zhang X., Zhou H.Y., Yang X., Banerjee O., Acosta J.N., Miller J., Huang O., Rajpurkar P. (2024). ReXrank: A Public Leaderboard for AI-Powered Radiology Report Generation. arXiv.

[8] Obuchowicz R., Lasek J., Wodziński M., Piórkowski A., Strzelecki M., Nurzynska K. (2025). Artificial Intelligence-Empowered Radiology–Current Status and Critical Review. Diagnostics.

[9] Park J., Oh K., Han K., Lee Y.H. (2024). Patient-centered radiology reports with generative artificial intelligence: Adding value to radiology reporting. Sci. Rep..

[10] Sloan P., Clatworthy P., Simpson E., Mirmehdi M. (2024). Automated Radiology Report Generation: A review of recent advances. IEEE Rev. Biomed. Eng..

[11] Dhamanskar P., Thacker C. (2024). A detailed analysis of deep learning-based techniques for automated radiology report generation. Int. J. Power Electron. Drive Syst. J. Electr. Comput. Eng..

[12] Reale-Nosei G., Amador-Domínguez E., Serrano E. (2024). From vision to text: A comprehensive review of natural image captioning in medical diagnosis and radiology report generation. Med Image Anal..

[13] Soleimani M., Seyyedi N., Ayyoubzadeh S.M., Kalhori S.R.N., Keshavarz H. (2024). Practical evaluation of CHATGPT performance for Radiology Report Generation. Acad. Radiol..

[14] Liu C., Tian Y., Chen W., Song Y., Zhang Y. (2024). Bootstrapping large language models for radiology report generation. Proc. AAAI Conf. Artif. Intell..

[15] Jeblick K., Schachtner B., Dexl J., Mittermeier A., Stüber A.T., Topalis J., Weber T., Wesp P., Sabel B.O., Ricke J. (2023). ChatGPT makes medicine easy to swallow: An exploratory case study on simplified radiology reports. Eur. Radiol..

[16] Ma C., Wu Z., Wang J., Xu S., Wei Y., Liu Z., Zeng F., Jiang X., Guo L., Cai X. (2024). An iterative Optimizing Framework for radiology report summarization with ChatGPT. IEEE Trans. Artif. Intell..

[17] Rashed E.A., Hussain W., Mousa M., al Shatouri M. Automatic Generation of Brain Tumor Diagnostic Reports from Multimodality MRI Using Large Language Models. Proceedings of the 2025 IEEE 22nd International Symposium on Biomedical Imaging (ISBI).

[18] Khoriba G., Nouman M., Rashed E.A. (2025). Large Language Models in Medical Image Understanding. Cutting-Edge Artificial Intelligence Advances and Implications in Real-World Applications.

[19] Dimensions AI. https://www.dimensions.ai/.

[20] Wang Z., Liu L., Wang L., Zhou L. METransformer: Radiology Report Generation by Transformer with Multiple Learnable Expert Tokens. Proceedings of the 2022 IEEE/CVF Conference on Computer Vision and Pattern Recognition (CVPR).

[21] Wang Z., Tang M., Wang L., Li X., Zhou L. (2022). A Medical Semantic-Assisted Transformer for Radiographic Report Generation.

[22] Huang Z., Zhang X., Zhang S. KIUT: Knowledge-injected U-Transformer for Radiology Report Generation. Proceedings of the 2022 IEEE/CVF Conference on Computer Vision and Pattern Recognition (CVPR).

[23] Hamamci I.E., Er S., Menze B. (2024). CT2REP: Automated Radiology Report Generation for 3D Medical Imaging.

[24] Sîrbu I., Sîrbu I.R., Bogojeska J., Rebedea T. (2025). GIT-CXR: End-to-End transformer for Chest X-Ray Report Generation. arXiv.

[25] Wang J., Yang Z., Hu X., Li L., Lin K., Gan Z., Liu Z., Liu C., Wang L. (2022). GIT: A Generative Image-to-text Transformer for Vision and Language. arXiv.

[26] Liu F., Wu X., Ge S., Fan W., Zou Y. Exploring and distilling posterior and prior knowledge for radiology report generation. Proceedings of the 2022 IEEE/CVF Conference on Computer Vision and Pattern Recognition (CVPR).

[27] Alqahtani F.F., Mohsan M.M., Alshamrani K., Zeb J., Alhamami S., Alqarni D. (2024). CNX-B2: A novel CNN-Transformer approach for chest X-Ray Medical Report Generation. IEEE Access.

[28] Gao D., Kong M., Zhao Y., Huang J., Huang Z., Kuang K., Wu F., Zhu Q. (2023). Simulating doctors’ thinking logic for chest X-ray report generation via Transformer-based Semantic Query learning. Med. Image Anal..

[29] Cao Y., Cui L., Zhang L., Yu F., Li Z., Xu Y. (2023). MMTN: Multi-Modal Memory Transformer Network for Image-Report Consistent Medical Report Generation. Proc. AAAI Conf. Artif. Intell..

[30] Tanida T., Müller P., Kaissis G., Rueckert D. Interactive and explainable region-guided radiology report generation. Proceedings of the 2022 IEEE/CVF Conference on Computer Vision and Pattern Recognition (CVPR).

[31] Onakpojeruo E.P., Mustapha M.T., Ozsahin D.U., Ozsahin I. (2024). A Comparative Analysis of the Novel Conditional Deep Convolutional Neural Network Model, Using Conditional Deep Convolutional Generative Adversarial Network-Generated Synthetic and Augmented Brain Tumor Datasets for Image Classification. Brain Sci..

[32] Lang W., Liu Z., Zhang Y. (2025). DACG: Dual Attention and Context Guidance model for radiology report generation. Med. Image Anal..

[33] Liu F., Yin C., Wu X., Ge S., Zhang P., Sun X. (2021). Contrastive Attention for Automatic Chest X-ray Report Generation. Findings of the Association for Computational Linguistics: ACL-IJCNLP 2021.

[34] Zhou Y., Huang L., Zhou T., Fu H., Shao L. Visual-Textual Attentive Semantic consistency for medical report generation. Proceedings of the 2021 IEEE/CVF International Conference on Computer Vision (ICCV).

[35] Tsaniya H., Fatichah C., Suciati N. (2024). Automatic radiology report generator using transformer with contrast-based image enhancement. IEEE Access.

[36] Onakpojeruo E., Mustapha M., Uzun Ozsahin D., Ozsahin I. (2024). Enhanced MRI-based brain tumor classification with a novel Pix2pix generative adversarial network augmentation framework. Brain Commun..

[37] Fan K., Cai X., Niranjan M. (2023). IIHT: Medical Report Generation with Image-to-Indicator Hierarchical Transformer.

[38] Voinea S.V., Mamuleanu M., Teica R.V., Florescu L.M., Selișteanu D., Gheonea I.A. (2024). GPT-Driven Radiology Report Generation with Fine-Tuned Llama 3. Bioengineering.

[39] Pellegrini C., Özsoy E., Busam B., Navab N., Keicher M. (2023). RADialog: A large Vision-Language model for radiology report generation and conversational assistance. arXiv.

[40] Zhang S., Sambara S., Banerjee O., Acosta J., Fahrner L.J., Rajpurkar P. (2024). RadFlag: A Black-Box Hallucination Detection Method for Medical Vision Language Models. arXiv.

[41] Udomlapsakul K., Pengpun P., Saengja T., Veerakanjana K., Tiankanon K., Khlaisamniang P., Supholkhan P., Chinkamol A., Aussavavirojekul P., Phimsiri H. SICAR at RRG2024: GPU Poor’s Guide to Radiology Report Generation. Proceedings of the 23rd Workshop on Biomedical Natural Language Processing.

[42] Li Y., Wang Z., Liu Y., Wang L., Liu L., Zhou L. (2024). KARGEN: Knowledge-Enhanced Automated Radiology Report Generation Using Large Language Models.

[43] Srivastav S., Ranjit M., Pérez-García F., Bouzid K., Bannur S., Castro D.C., Schwaighofer A., Sharma H., Ilse M., Salvatelli V. (2024). MAIRA at RRG24: A Specialised Large Multimodal Model for Radiology Report Generation.

[44] Wang F., Du S., Yu L. (2024). HERGen: Elevating Radiology Report Generation with Longitudinal Data. arXiv.

[45] Alkhaldi A., Alnajim R., Alabdullatef L., Alyahya R., Chen J., Zhu D., Alsinan A., Elhoseiny M. (2024). MiniGPT-MeD: Large Language Model as a General Interface for Radiology Diagnosis. arXiv.

[46] Kapadnis M.N., Patnaik S., Nandy A., Ray S., Goyal P., Sheet D. (2024). SERPENT-VLM: Self-Refining Radiology Report Generation using Vision Language Models. arXiv.

[47] Campanini D., Loch O., Messina P., Elberg R., Parra D. iHealth-Chile-1 at RRG24: In-context Learning and Finetuning of a Large Multimodal Model for Radiology Report Generation. Proceedings of the 23rd Workshop on Biomedical Natural Language Processing.

[48] Hou W., Cheng Y., Xu K., Li H., Hu Y., Li W., Liu J. (2025). RADAR: Enhancing Radiology Report Generation with Supplementary Knowledge Injection. arXiv.

[49] Zhang D., Ren A., Liang J., Liu Q., Wang H., Ma Y. (2022). Improving medical X-ray report generation by using Knowledge Graph. Appl. Sci..

[50] Xiang Z., Cui S., Shang C., Jiang J., Zhang L. (2024). GMoD: Graph-Driven Momentum Distillation Framework with Active Perception of Disease Severity for Radiology Report Generation.

[51] Liang X., Zhang Y., Wang D., Zhong H., Li R., Wang Q. (2024). Divide and Conquer: Isolating Normal-Abnormal Attributes in Knowledge Graph-Enhanced Radiology Report Generation. MM ’24: Proceedings of the 32nd ACM International Conference on Multimedia.

[52] Kale K., Bhattacharyya P., Gune M., Shetty A., Lawyer R. KGVL-BART: Knowledge Graph Augmented Visual Language BART for Radiology Report Generation. Proceedings of the 17th Conference of the European Chapter of the Association for Computational Linguistics.

[53] Kale K., Bhattacharyya P., Shetty A., Gune M., Shrivastava K., Lawyer R., Biswas S. (2023). “Knowledge is Power”: Constructing Knowledge Graph of Abdominal Organs and Using Them for Automatic Radiology Report Generation. Proceedings of the 61st Annual Meeting of the Association for Computational Linguistics (Volume 5: Industry Track).

[54] Xiong Y., Liu J., Zaripova K., Sharifzadeh S., Keicher M., Navab N. (2024). Prior-RadGraphFormer: A Prior-Knowledge-Enhanced Transformer for Generating Radiology Graphs from X-Rays.

[55] Hou X., Liu Z., Li X., Li X., Sang S., Zhang Y. (2023). MKCL: Medical Knowledge with Contrastive Learning model for radiology report generation. J. Biomed. Inform..

[56] Yin H., Wu W., Hao Y. (2024). DKA-RG: Disease-Knowledge-Enhanced Fine-Grained Image–Text Alignment for Automatic Radiology Report Generation. Electronics.

[57] Kang B., Xiong Y., Jiao J., Zhang Y., Jia X., Li J. Bridging the gap: Cross-modal knowledge driven network for radiology report generation. Proceedings of the 2023 IEEE International Conference on Bioinformatics and Biomedicine (BIBM).

[58] Zhang Y., Wang X., Xu Z., Yu Q., Yuille A., Xu D. (2020). When Radiology Report Generation Meets Knowledge Graph. arXiv.

[59] Zhang X., Acosta J.N., Zhou H.Y., Rajpurkar P. (2024). Uncovering Knowledge Gaps in Radiology Report Generation Models through Knowledge Graphs. arXiv.

[60] Li H., Zhu G., Zhang L., Jiang Y., Dang Y., Hou H., Shen P., Zhao X., Shah S.A.A., Bennamoun M. (2023). Scene Graph Generation: A comprehensive survey. Neurocomputing.

[61] Johnson J., Krishna R., Stark M., Li L.J., Shamma D.A., Bernstein M.S., Fei-Fei L. Image retrieval using scene graphs. Proceedings of the 2015 IEEE Conference on Computer Vision and Pattern Recognition (CVPR).

[62] Zhang K., Yang Y., Yu J., Fan J., Jiang H., Huang Q., Han W. (2024). Attribute prototype-guided iterative scene graph for explainable radiology report generation. IEEE Trans. Med. Imaging.

[63] Li R., Zhang S., Lin D., Chen K., He X. (2024). From Pixels to Graphs: Open-Vocabulary Scene Graph Generation with Vision-Language Models. arXiv.

[64] Wang J., Zhu L., Bhalerao A., He Y. (2024). Scene graph aided radiology report generation. arXiv.

[65] Özsoy E., Czempiel T., Örnek E.P., Eck U., Tombari F., Navab N. (2023). Holistic OR domain modeling: A semantic scene graph approach. Int. J. Comput. Assist. Radiol. Surg..

[66] Özsoy E., Örnek E.P., Eck U., Tombari F., Navab N. (2021). Multimodal semantic scene graphs for holistic modeling of surgical procedures. arXiv.

[67] Pelka O., Koitka S., Rückert J., Nensa F., Friedrich C.M. (2018). Radiology Objects in COntext (ROCO): A Multimodal Image Dataset.

[68] Demner-Fushman D., Kohli M.D., Rosenman M.B., Shooshan S.E., Rodriguez L., Antani S., Thoma G.R., McDonald C.J. (2015). Preparing a collection of radiology examinations for distribution and retrieval. J. Am. Med Inform. Assoc..

[69] Bustos A., Pertusa A., Salinas J.M., De La Iglesia-Vayá M. (2020). PadChest: A large chest x-ray image dataset with multi-label annotated reports. Med. Image Anal..

[70] Johnson A.E.W., Pollard T.J., Berkowitz S.J., Greenbaum N.R., Lungren M.P., Deng C.Y., Mark R.G., Horng S. (2019). MIMIC-CXR, a de-identified publicly available database of chest radiographs with free-text reports. Sci. Data.

[71] Johnson A.E.W., Pollard T.J., Greenbaum N.R., Lungren M.P., Deng C.Y., Peng Y., Lu Z., Mark R.G., Berkowitz S.J., Horng S. (2019). MIMIC-CXR-JPG, a large publicly available database of labeled chest radiographs. arXiv.

[72] Wang F., Liang X., Xu L. (2021). Unifying relational sentence generation and retrieval for medical image report composition. arXiv.

[73] Li M., Wang F., Chang X., Liang X. (2020). Auxiliary Signal-Guided Knowledge Encoder-Decoder for medical report generation. arXiv.

[74] Wu J.T., Agu N.N., Lourentzou I., Sharma A., Paguio J.A., Yao J.S., Dee E.C., Mitchell W., Kashyap S., Giovannini A. (2021). Chest IMAGenome Dataset for clinical reasoning. arXiv.

[75] Nishino T., Miura Y., Taniguchi T., Ohkuma T., Suzuki Y., Kido S., Tomiyama N. (2022). Factual Accuracy is not Enough: Planning Consistent Description Order for Radiology Report Generation. Proceedings of the 2021 Conference on Empirical Methods in Natural Language Processing.

[76] Boecking B., Usuyama N., Bannur S., Castro D.C., Schwaighofer A., Hyland S., Wetscherek M., Naumann T., Nori A., Alvarez-Valle J. (2022). Making the Most of Text Semantics to Improve Biomedical Vision–Language Processing.

[77] Ramesh V., Chi N.A., Rajpurkar P. (2022). Improving radiology report generation systems by removing hallucinated references to non-existent priors. arXiv.

[78] Hamamci I.E., Er S., Almas F., Simsek A.G., Esirgun S.N., Dogan I., Dasdelen M.F., Wittmann B., Simsar E., Simsar M. (2024). A foundation model utilizing chest CT volumes and radiology reports for supervised-level zero-shot detection of abnormalities. arXiv.

[79] Metmer H., Yang X. (2024). An open chest X-ray dataset with benchmarks for automatic radiology report generation in French. Neurocomputing.

[80] Papineni K., Roukos S., Ward T., Zhu W.J. BLEU. Proceedings of the 40th Annual Meeting of the Association for Computational Linguistics.

[81] Lin C.Y. ROUGE: A Package for Automatic Evaluation of Summaries. Proceedings of the Text Summarization Branches Out.

[82] Denkowski M., Lavie A. Meteor 1.3: Automatic Metric for Reliable Optimization and Evaluation of Machine Translation Systems. Proceedings of the Sixth Workshop on Statistical Machine Translation.

[83] Zhang T., Kishore V., Wu F., Weinberger K.Q., Artzi Y. (2019). BERTScore: Evaluating Text Generation with BERT. arXiv.

[84] Vedantam R., Zitnick C.L., Parikh D. (2014). CIDER: Consensus-based Image Description Evaluation. arXiv.

[85] Chen Z., Song Y., Chang T.H., Wan X. (2020). Generating Radiology Reports via Memory-driven Transformer. Proceedings of the 2020 Conference on Empirical Methods in Natural Language Processing (EMNLP).

[86] Irvin J., Rajpurkar P., Ko M., Yu Y., Ciurea-Ilcus S., Chute C., Marklund H., Haghgoo B., Ball R., Shpanskaya K. (2019). CheXpert: A Large Chest Radiograph Dataset with Uncertainty Labels and Expert Comparison. Proc. AAAI Conf. Artif. Intell..

[87] Endo M., Krishnan R., Krishna V., Ng A.Y., Rajpurkar P. Retrieval-Based Chest X-Ray Report Generation Using a Pre-trained Contrastive Language-Image Model. Proceedings of the Machine Learning for Health. PMLR.

[88] Yu F., Endo M., Krishnan R., Pan I., Tsai A., Reis E.P., Fonseca E.K.U.N., Lee H.M.H., Abad Z.S.H., Ng A.Y. (2023). Evaluating progress in automatic chest X-ray radiology report generation. Patterns.

[89] Langlotz C.P. (2006). RadLex: A new method for indexing online educational materials. Radiographics.

[90] Jain S., Agrawal A., Saporta A., Truong S.Q., Duong D.N., Bui T., Chambon P., Zhang Y., Lungren M.P., Ng A.Y. (2021). RadGraph: Extracting Clinical Entities and Relations from Radiology Reports. arXiv.

[91] Liu Y., Wang Z., Li Y., Liang X., Liu L., Wang L., Zhou L. (2024). MRScore: Evaluating Radiology Report Generation with LLM-based Reward System. arXiv.

[92] Huang A., Banerjee O., Wu K., Reis E.P., Rajpurkar P. (2024). FineRadScore: A Radiology Report Line-by-Line Evaluation Technique Generating Corrections with Severity Scores. arXiv.

